# *Toxoplasma gondii* infection and toxoplasmosis in farm animals: Risk factors and economic impact

**DOI:** 10.1016/j.fawpar.2019.e00037

**Published:** 2019-04-03

**Authors:** S. Stelzer, W. Basso, J. Benavides Silván, L.M. Ortega-Mora, P. Maksimov, J. Gethmann, F.J. Conraths, G. Schares

**Affiliations:** a Friedrich-Loeffler-Institut, Federal Research Institute for Animal Health, Institute of Epidemiology, Südufer 10, 17493 Greifswald - Insel Riems, Germany; b Institute of Parasitology, University of Bern, Länggassstrasse 122, 3012 Bern, Switzerland; c Instituto de Ganadería de Montaña (CSIC-Universidad de León) Grulleros, 24346 León, Spain; d SALUVET, Animal Health Department, Faculty of Veterinary Sciences, Complutense University of Madrid, Ciudad Universitaria s/n, 28040 Madrid, Spain

**Keywords:** Zoonosis, Livestock, Prevalence, Natural infection, Experimental infection, Costs

## Abstract

The protozoan parasite *Toxoplasma gondii* is a zoonotic parasite that can be transmitted from animals to humans. Felids, including domestic cats, are definitive hosts that can shed oocysts with their feces. In addition to infections that occur by accidental oral uptake of food or water contaminated with oocysts, it is assumed that a large proportion of affected humans may have become infected by consuming meat or other animal products that contained infective parasitic stages of *T. gondii*. Since farm animals represent a direct source of infection for humans, but also a possible reservoir for the parasite, it is important to control *T. gondii* infections in livestock. Moreover, *T. gondii* may also be pathogenic to livestock where it could be responsible for considerable economic losses in some regions and particular farming systems, e.g. in areas where the small ruminant industry is relevant.

This review aims to summarize actual knowledge on the prevalence and effects of infections with *T. gondii* in the most important livestock species and on the effects of toxoplasmosis on livestock. It also provides an overview on potential risk factors favoring infections of livestock with *T. gondii*. Knowledge on potential risk factors is prerequisite to implement effective biosecurity measures on farms to prevent *T. gondii* infections. Risk factors identified by many studies are cat-related, but also those associated with a potential contamination of fodder or water, and with access to a potentially contaminated environment. Published information on the costs *T. gondii* infections cause in livestock production, is scarce. The most recent peer reviewed reports from Great Britain and Uruguay suggest annual cost of about 5–15 million US $ per country. Since these estimates are outdated, future studies are needed to estimate the present costs due to toxoplasmosis in livestock. Further, the fact that *T. gondii* infections in livestock may affect human health needs to be considered and the respective costs should also be estimated, but this is beyond the scope of this article.

## Highlights

• Brief overview on *T. gondii* prevalence in livestock
• Natural and experimental *T. gondii* infections
• Risk factors for *T. gondii* infection of livestock
• Economic impact of toxoplasmosis in livestock

## Graphical abstract

**Figure:**
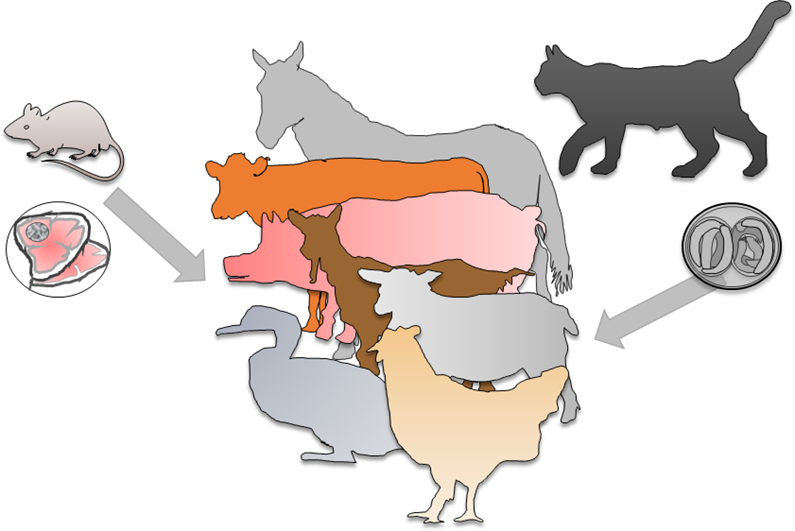

## 1 Introduction

*Toxoplasma gondii* is a zoonotic apicomplexan parasite that is able to infect probably all warm-blooded animals, including livestock (Dubey, 2010b; Schlüter et al., 2014). Domestic cats and other felids are definitive hosts of *T. gondii*. This implies that the parasite is only able to complete its sexual life cycle in these species, i.e. environmentally resistant oocysts can only be shed with the feces of infected felids (Dubey, 2010b). Oocysts are central in the life cycle of *T. gondii*. After one up to a few days of maturation (sporulation) in the environment, they become infective to a large variety of warm-blooded intermediate hosts (livestock, synanthropic and wild living animals such as rodents or birds, poultry or humans) if ingested (Dubey, 2010b). In addition to oocysts, there are two further stages of *T. gondii*, which are infective, i.e. tachyzoites and bradyzoites, the latter being present in tissue cysts. After infection, tachyzoites invade host cells, in which they multiply. This replication is strictly intracytoplasmatic in parasitophorous vacuoles formed by the parasite (Schlüter et al., 2014). In parallel, after several rounds of multiplication, the parasite establishes intracellular tissue cysts, which contain slowly or no longer replicating bradyzoites (Jerome et al., 1998; Radke et al., 2003; Watts et al., 2015). Tissue cyst formation preferentially occurs in brain tissue, the skeletal and heart muscle or also in the retina of infected intermediate hosts (Schlüter et al., 2014).

While the tachyzoite stages are repressed after the onset of the immune response of the host, the dormant tissue cysts ensure that *T. gondii* infections persist inside the host cells, where they are protected from the immune system, possibly for the rest of the life of the intermediate host (Rougier et al., 2017). Tissue cysts may contain hundreds of bradyzoites (Dubey, 2010b). If carnivorous or omnivorous animals feed on material that contains tissue cysts, encysted bradyzoites can survive the gastric passage and initiate parasite multiplication in the intestine of the infected intermediate or definitive host (Dubey, 2010b). In some regions of the world, in particular in Europe, risk factor studies suggested that most humans become infected by ingesting bradyzoites present in undercooked or not sufficiently inactivated meat (Cook et al., 2000; Kapperud et al., 1996).

*Toxoplasma gondii* is transmitted vertically in several intermediate host species including humans (Dubey, 2010b). The transplacental vertical transmission is facilitated by tachyzoites usually after the primary and during the acute phase of infection (Dubey, 2010b). Tachyzoites circulating after a reactivated chronic infection also seem to be able to facilitate vertical transmission in some livestock species, although experimental findings suggest that this might be a rare event in *T. gondii* (Dubey, 1982).

As farm animals represent a source of infection for humans and reservoirs of *T. gondii* for wildlife it has been proposed to reduce *T. gondii* infections in livestock as much as possible, particularly in pigs (Anonymous, 2011a, Anonymous, 2011b). Potential risk factors for *T. gondii* infections in livestock have been studied and were reviewed in recent years (Guo et al., 2015; Opsteegh et al., 2016), but since the knowledge on the epidemiology of toxoplasmosis in animals and humans is rapidly evolving, these reviews deserved an update. Therefore, the main objectives of this study were (i) to briefly summarize the recent gain in knowledge on the prevalence of *T. gondii* in the most important livestock species and on the importance of infection with this parasite in livestock production, (ii) to compile existing knowledge on the effect of natural and experimental *T. gondii* infections in the dominant livestock species, and finally (iii) to provide an up-to-date summary on potential risk factors and risk factor studies for *T. gondii* infection in livestock.

## 2 *Toxoplasma gondii* infections in livestock animals – importance for livestock production and prevalence

### 2.1 Pigs

#### 2.1.1 Prevalence in pigs

Seroepidemiological surveys have provided evidence for a worldwide distribution of *T. gondii* in pigs, with prevalences varying according to age, pig categories, geography and management system. In general, a low prevalence of *T. gondii* infections (<1%) is observed in pigs reared in confinement with controlled management conditions, preventing access of rodents and cats, whereas higher prevalence values (>60%) can be found in farms with free-ranging pigs, farms without controlled conditions allowing outdoor access and in backyard holdings (De Berardinis et al., 2017).

Worldwide information on *T. gondii* infection in pigs up to 2009 was reviewed several times in the past (Dubey, 1986b, Dubey, 2009a; Dubey and Beattie, 1988). The situation in the USA was also reviewed more recently (Hill and Dubey, 2013). A systematic review, based on reports on a direct identification of *T. gondii* in pigs and pork identified a pooled *T. gondii* prevalence of 12.3% with a 95% prediction interval ranging from 0 to 55% (Belluco et al., 2017). In Europe, the seroprevalence for *T. gondii*-specific antibodies was reported to range from 0 to 64% in fattening pigs and from 3 to 31% in breeding sows (De Berardinis et al., 2017). In Africa, a systematic review and meta-data analysis of seroepidemiological studies reported seroprevalences from 9.3 to 40.6%, with an overall estimated *T. gondii* seroprevalence in pigs of 26% (Tonouhewa et al., 2017). In China, pigs are assumed to represent one of the farm animal species most frequently infected with *T. gondii*. It was estimated that 30 to 50% of the raised pigs are seropositive, reaching a prevalence of 70% in some areas and farms (Pan et al., 2017). There is quite a large number of further and more recent local studies on prevalence in pigs, but there is little information in addition to that what has been reported and summarized before.

#### 2.1.2 Possible routes of infection in pigs

Most *T. gondii* infections in pigs are acquired postnatally, either by ingestion of sporulated oocysts in contaminated soil, feed and water, or by ingestion of cysts in the tissues of infected intermediate hosts (e.g. rodents, birds, meat and cannibalism). Pigs can also become infected prenatally by transplacental transmission of the parasite (Dubey, 2009a). The occurrence of galactogenic transmission of *T. gondii* from the sow to the piglets has been reported, but might be a rare event (Basso et al., 2017). It is presumed that infection through oocysts accounts for most infections in conventional pig breeding systems and especially in outbreaks of clinical toxoplasmosis involving several animals (Kim et al., 2009; Li et al., 2010; Okamoto et al., 1989; Weissenbock and Dubey, 1993).

#### 2.1.3 Disease caused by *T. gondii* infections in pigs

*Toxoplasma gondii* infections in pigs are commonly subclinical; nevertheless, several cases of clinical disease after natural infection have been recorded worldwide (Dubey, 1986b, Dubey, 2009a; Dubey and Beattie, 1988). Clinical manifestations seem to occur more frequently in neonatal and weaned pigs, but also cases of clinical toxoplasmosis affecting sows have been described. Common signs observed in clinically infected pigs include anorexia, apathy, fever, ocular and nasal discharge, dyspnoea, cyanosis, diarrhea, limb weakness, neurological signs and sometimes death (Dubey, 1986b, Dubey, 2009a; Dubey and Beattie, 1988). None of these signs is pathognomonic for toxoplasmosis. Besides, *T. gondii* infections may be associated with reproductive failure in sows characterized by abortion, fetal mummification, stillbirth and neonatal mortality (Basso et al., 2015; Dubey, 1986b, Dubey, 2009a; Dubey and Beattie, 1988). Clinical disease is believed to occur only during the acute phase of infection as result of necrotic and inflammatory processes during tachyzoite multiplication in several tissues. Chronically infected animals do not have clinical signs, but they represent an important source of infection for humans, in particular, if undercooked pork or insufficiently treated meat products containing tissue cysts are consumed (Dubey, 2009a). Different factors are believed to influence the clinical presentation of *T. gondii* infection in pigs such as age and immune status of the host, co-infection with other agents, the parasitic stage of *T. gondii* (i.e. oocyst, tissue cyst), infection dose, and the strain or the genetic background of *T. gondii*. In some cases, viral infections such as Porcine Circovirus type 2 (Klein et al., 2010) and Porcine Parvovirus (Basso et al., 2015) were also associated with clinical manifestations of toxoplasmosis in pigs.

Reports prior to 2009 were summarized previously (Dubey, 1986b, Dubey, 2009a; Dubey and Beattie, 1988). Since then, further confirmed cases of clinical toxoplasmosis involving (i) suckling piglets in Brazil (Olinda et al., 2016), (ii) fattening pigs in China (Li et al., 2010), Germany (Klein et al., 2010) and Brazil (Olinda et al., 2016) and (iii) *T. gondii* infections associated with reproduction failure in sows in Switzerland (Basso et al., 2015) were published.

A severe outbreak of toxoplasmosis in fattening pigs was reported from Gansu Province, China, with morbidity affecting 549/960 (57%) fattening pigs (Li et al., 2010). The pigs had fever (40–42.2 °C), anorexia and depression, and 19 of the affected pigs died. Serological analysis of 154 clinically ill animals had *T. gondii* IgG or IgM positive ELISA results in 142 (92.2%) and 147 (95.4%) of the animals, respectively. Moreover, *T. gondii* could be isolated in mice by intraperitoneal inoculation of pooled heart, liver, spleen and brain tissues from two pigs which showed clinical signs. The source of infection was assumed to be feed contaminated with cat feces. A controlled feeding experiment administering randomly collected feed to five seronegative piglets lead to development of fever, depression and seroconversion of three of the animals. In another study (Klein et al., 2010), systemic toxoplasmosis was diagnosed in a 3.5-month-old fattening pig suffering from post-weaning multisystemic wasting syndrome associated with PCV-2 infection in Germany. The pig had severe respiratory signs and died suddenly. Immunohistochemically, *T. gondii* was detected associated with interstitial and necrotizing pneumonia, lymphadenitis and adrenal necrosis. It was assumed that immunosuppression caused by primary PCV-2 infection may have triggered secondary systemic toxoplasmosis (Klein et al., 2010).

Interestingly, many of these cases of clinical toxoplasmosis in pigs were registered in Asia (i.e. Japan, Taiwan, China, Korea, Thailand), although there are reports of clinical toxoplasmosis in pigs from several countries around the world (Dubey, 2009a; Dubey and Beattie, 1988). It is not known if specific *T. gondii* genotypes circulating in Asia may be more prone to cause clinical infections in pigs (Basso et al., 2017).

Recently, *T. gondii* parasites belonging to the Chinese 1 genotype (synonymous to ToxoDB#9), a frequent genotype in Asia and especially in China (Hou et al., 2018; Wang et al., 2017), were detected in apparently independent fatal cases of toxoplasmosis in two pigs in Brazil (Olinda et al., 2016). The animals (aged one and four months) showed apathy, dyspnoea, poor general condition and died after a few days. The main lesions in both pigs consisted of severe diffuse necrotizing bronchointerstitial pneumonia associated with numerous *T. gondii* tachyzoites present in the lesions. Interestingly, the cases occurred 3 months apart from each other and both animals derived from two different farms, showing that *T. gondii* resembling a ToxoDB#9-like genotype is circulating in Brazil (Olinda et al., 2016). This genotype was also identified in 16 out of 17 samples from infected pigs with high fever, dyspnoea, subcutaneous haemorrhage, abortion, enlargement and necrosis of liver and spleen suspected of having clinical toxoplasmosis in China (Jiang et al., 2013). In China, *T. gondii* infections in pigs are very common and outbreaks of clinical toxoplasmosis with death of numerous pigs have been reported on several occasions (summarized by Pan et al. (2017) and Li et al. (2010)). Moreover, there are reports of repeated outbreaks over 5 years in an individual pig farm in the Shandong Province (Li et al., 2010). These outbreaks of fatal toxoplasmosis were thought to be related to consumption of feed contaminated with oocysts from cat feces (Li et al., 2010). Unfortunately, no molecular characterization of the isolates involved in these outbreaks was performed. Studies in Jiangsu province, Eastern China, revealed high positive rates of *T. gondii* infection in sick pigs (showing poor mental state, fever, and/or dyspnoea) with 46.8% (66/141) PCR positive animals in various tissues (Hou et al., 2018). In 58 pigs, coinfection with other pathogens was observed but in seven animals *T. gondii* was the only agent detected, suggesting that it could be involved in the aetiology of sickness or death of pigs in that region. Molecular analysis of *T. gondii* from 17 sick pigs showed that *T. gondii* Chinese 1 (ToxoDB#9) was the most frequently (11/17) detected genotype (Hou et al., 2018). In China, parasites with this genotype were also isolated from one case of human toxoplasmosis, but in several American countries also from subclinically infected livestock animals (summarized in Olinda et al. (2016)).

Contrary to the vast knowledge about the importance of vertical transmission of *T. gondii* in small ruminants and humans, the role of *T. gondii* as cause of reproductive disorders in sows and the epidemiological significance of intrauterine and galactogenic infections in piglets, showing no clinical signs are less understood (Basso et al., 2017). Reports of reproductive failure due to toxoplasmosis and congenital infection in piglets are well documented, but the experimental reproduction of vertical transmission in pregnant sows is often not successful (Basso et al., 2017). In general, sows that abort or deliver infected offspring usually do not show further clinical signs, but fever, anorexia, neurological signs and even death were observed on some occasions in sows that aborted and transmitted the infection to the fetuses (Kim et al., 2009). In China, abortions caused by *T. gondii* in sows are considered common and assumed to cause economic losses (Pan et al., 2017). In Europe, reports of reproductive problems due to *T. gondii* infection in pigs are scarce. A large epidemiological study in 94 pig breeding farms in Germany suggested an association of *T. gondii* with reproductive failure in sows. The within-farm seroprevalence to *T. gondii* was significantly higher in farms experiencing reproductive disorders (repeat-breeders, abortion, neonatal mortality), than in farms without such problems, but the role of *T. gondii* in causing these reproductive problems was not further assessed (Damriyasa et al., 2004). Recently, *T. gondii* was detected in the placenta or in fetuses of 34 out of 113 sows that had aborted or delivered a high number of stillborn or weak piglets in Switzerland (Basso et al., 2015). By real time PCR, *T. gondii* DNA was detected in three placentas from one seropositive sow (abortion at 71 days of gestation [dg]), and in brain tissues from one fetus (abortion at 76 dg), one stillborn (116 dg) and one mummy (112 dg) originating from three further seropositive sows, but in no sample derived from the seronegative dams (Basso et al., 2015). By contrast, the examination of fetal tissues and fluids from 32 sow abortions in Romania by PCR did not yield any *T. gondii* positive samples (Iovu et al., 2010).

#### 2.1.4 Effects of experimental infections in pigs

Pigs can be experimentally infected with any *T. gondii* stage (i.e. oocysts, tissue cysts, tachyzoites). Most experimentally inoculated pigs, including animals inoculated with very low infection doses (as few as 1 or 10 oocysts), seroconverted after 2–4 weeks and the parasite could be successfully recovered from different tissues. However, experimental reproduction of clinical toxoplasmosis, vertical transmission and congenital toxoplasmosis in pigs is considered difficult (Boch et al., 1965b; Dubey, 2009a; Dubey and Beattie, 1988; Dubey and Urban, 1990; Moller et al., 1970; Sanger and Cole, 1955; Work et al., 1970). Various parasite related factors (i.e. *T. gondii* stage, dose, infection route, virulence and the genetic background of the strain) and host related factors (i.e. breed, age, immune status and stage of gestation) may influence the outcome of an experimental infection (Dubey, 1986b, Dubey, 2009a). Weaned pigs fed oocysts or tissue cysts often developed transient clinical signs such as weight loss, anorexia and/or fever, independent of the *T. gondii* isolate in the inoculum and generally recovered by three weeks post inoculation (Basso et al., 2013; Dubey, 2009a).

Experimental infections with *T. gondii* in pigs were performed within the framework of numerous studies aiming to reveal different aspects of the biology of the parasite (pathogenesis, immune response, persistence of the infection in the tissues, reproduction of congenital toxoplasmosis, development and evaluation of diagnostic methods) or aiming to establish vaccines (Dubey, 2009a; Dubey and Beattie, 1988).

It seems that clinical toxoplasmosis in any pig category and vertical transmission of *T. gondii* in pregnant sows can be more frequently reproduced by intravenous inoculation of high doses of tachyzoites than by feeding tissue cysts or oocysts. Furthermore, the potential occurrence of vertical transmission may be influenced by the *T. gondii* isolate used in the inoculations (Basso et al., 2017; Dubey and Urban, 1990; Jungersen et al., 2001; Work et al., 1970). Oral inoculations with 10^3^ oocysts of the GT-1 strain (Type I strain; ToxoDB#10) led to a transplacental infection in five out of 11 inoculated pregnant sows and to transient lethargy, anorexia and respiratory distress between 5 and 15 days post infection (dpi) (Dubey and Urban, 1990), while inoculations with 10^4^ to10^5^ oocysts of the CZ isolate (a European Type II isolate, Toxo DB#3) were not able to reproduce vertical transmission or other clinical signs in any of the 8 pregnant and infected sows (Basso et al., 2017). Likewise, feeding of 5 × 10^3^ oocysts of the CZ isolate to six 4.5 week-old piglets caused infection in all animals but only transiently fever (in all animals); apathy, anorexia and soft feces (in only one piglet) were observed, suggesting a low virulence of this isolate for pigs (Basso et al., 2013). Nevertheless, some authors considered low pathogenic *T. gondii* strains as good candidates to reproduce vertical transmissions in sows as these parasites might produce a subclinical infection in the dam, having a better chance of establishing placental infections and congenital toxoplasmosis in the piglets before development of a limiting immune response in the sow (Jungersen et al., 2001).

Experimental infections of pigs have recently been performed to evaluate viability of *T. gondii* in meat after processing techniques. Twelve pigs were inoculated with 10^3^ *T. gondii* oocysts of a type II field isolate from cat feces and slaughtered 4 months after inoculation. Clinical signs were not reported, but the pigs seroconverted post inoculation and PCR positive results were obtained from most thighs, both at slaughter and post curing (Genchi et al., 2017). In two further experimental studies conducted to test vaccination or to assess swine as an experimental model for human ocular toxoplasmosis, no clinical signs and also no ocular toxoplasmosis were reported after experimental infection with either 10^3^ oocysts per animal or 10^3^ tissue cysts per animal of the M4 strain (a *T. gondii* Type II strain) of pigs (Burrells et al., 2015; Garcia et al., 2017).

### 2.2 Sheep and goats

Sheep and goats are highly susceptible for infections with *T. gondii* and this protozoan parasite is considered a major cause of reproductive losses in small ruminants worldwide. While most descriptions and investigations have been carried out in sheep (Dubey, 2009b), toxoplasmosis has a similar or even greater importance as an abortive disease in goats (Dubey, 2010b). In addition, toxoplasmosis is a relevant zoonosis and infection in small ruminants may play a major role in its transmission to humans (Belluco et al., 2016; Opsteegh et al., 2016).

#### 2.2.1 Prevalence in sheep and goats

Antibodies to *T. gondii* have been found in sheep and goats worldwide. More than 200 articles reported seroprevalence studies in these domestic ruminant species before 2010, as reviewed by Dubey, 2009b, Dubey, 2010b. At that time, areas of the world with a large number of seroprevalence reports were Brazil, Europe, North America, and the Middle East. From 2010 to 2018, further epidemiological studies in small ruminants have been published, including reports from areas where information was scarce and regions, where sheep and goats are relevant livestock species. These studies are from different parts of Asia (i.e. China, Pakistan, South East Asia), Sub-Saharan Africa and countries from the Mediterranean (Ahmed et al., 2016; Dong et al., 2018; Garcia-Bocanegra et al., 2013; Gazzonis et al., 2015; Kantzoura et al., 2013; Khan et al., 2017; Tilahun et al., 2018; Tzanidakis et al., 2012). Although differences in study design, purpose of the study, serological methodology and cut off points applied make it difficult or even impossible to compare data, these as well as the previous studies clearly show that *T. gondii* infections are highly prevalent in small ruminants (Dubey, 2010b). In the following, representative examples of recent studies conducted on different continents are summarized.

In Africa, in a recent meta-analysis, summarizing data from 1969 to 2016, the overall estimated prevalence was 26.1% (17.0–37.0%) for sheep and 22.9% (12.3–36.0%) for goats (Tonouhewa et al., 2017). In Egypt, antibody prevalence was higher in goats (62%) than in sheep (between 4.1 and 26%) (Al-Kappany et al., 2018). In Tunisia, antibodies to *T. gondii* were found in 40.2% sheep and 34.5% goats (Lahmar et al., 2015). In Ethiopia, the seroprevalence of *T. gondii* infection in sheep and goats was 33.7% and 27.6%, respectively (Tilahun et al., 2018). A further study from this country reported high flock (59.7%) and individual animal (31.8%) *T. gondii* seroprevalences associated with abortion in some districts (Gebremedhin et al., 2014). A lower seroprevalence was reported from South Africa with 8% in sheep (Hammond-Aryee et al., 2015).

In America, a systematic meta-analysis provided estimates on *T. gondii* infection in food animals produced in the United States, including small ruminants. *T. gondii* infection seroprevalence in goats (30.7%) was higher than in sheep or lambs (22.0%) (Guo et al., 2016). Further studies report *T. gondii* seroprevalences in sheep and goats from the Caribbean Islands Dominica (67%, 58%), Grenada (48%, 57%), Montserrat (89%, 80%) and St. Kitts and Nevis (57%, 42%) (Hamilton et al., 2014). In another study, antibodies to *T. gondii* (Modified Agglutination Test (MAT) titre 1:≥25) were found in 44.1% of sheep and 42.8% goats in Grenada and Carriacou (Chikweto et al., 2011). In Brazil, serum samples of 930 sheep were tested in two regions of Rio Grande do Norte (Northeastern Brazil), with different climatic conditions and the overall estimated prevalence was 22.1% (Andrade et al., 2013).

Regarding Asia, the situation of *T. gondii* infections has recently been reviewed for China. Seroprevalence for *T. gondii* in sheep has been estimated to be 11.8% (2305/19,565) and the overall estimated seroprevalence for *T. gondii* in goats was 17.6% (3260/18,556) (Dong et al., 2018). In Myanmar, an 11.4% seroprevalence has been reported in goats (Bawm et al., 2016). In other South Asian countries, reported prevalence in sheep and goats was 21.1% and 25.4%, respectively (Khan et al., 2017). In Pakistan, the results also showed higher seroprevalence of *T. gondii* in goats (42.8%) as compared to sheep (26.2%) (Ahmed et al., 2016). In Saudi Arabia, antibodies to *T. gondii* were found in 36.4% (325/891) of sheep and 35.3% (196/555) of goats (Alanazi, 2013).

In Europe, high prevalence values have been observed in both, sheep and goats in Mediterranean countries. In Greece, in one study, sheep had a higher seroprevalence (48.6% [729/1501]) for *T. gondii* than goats (30.7% [166/541]) (Tzanidakis et al., 2012). In Thessaly, a total of 540 sheep and goat serum samples were examined and the seroprevalence was 24.5% (Kantzoura et al., 2013). In another study, specific IgG against *T. gondii* were detected in 53.71% and 61.3% of the sheep and goats from mixed flocks (Diakoua et al., 2013). In Northern Italy, antibodies were found in 96.6% of goat farms and in 87.5% of sheep farms; 41.7% goats and 59.3% sheep had a positive result. The seroprevalence was significantly higher in sheep than in goats (Gazzonis et al., 2015). In Portugal, 33.6% of sheep and 18.5% of goats were seropositive by a modified agglutination test (MAT) (A.P. Lopes et al., 2013). In Southern Spain, 248 (49.3%) of 503 sheep, and 124 (25.1%) of 494 goats were seropositive. The herd seroprevalence was 84.7% (61/72), and 72.2% (52/72) for sheep and goats, respectively (Garcia-Bocanegra et al., 2013). In another study in the same region, the seroprevalence was 41.2% in sheep and 5.6% in goats (Almeria et al., 2018). In the northwestern part of Spain, individual (48%) and flock-level (74%) *T. gondii* seroprevalence values in goats were high; the within-flock prevalence was 53% (Diaz et al., 2016). In Eastern Europe as Poland, seroprevalences of 21% in goats and 47% in sheep have been reported (Moskwa et al., 2018). In Romania, the seroprevalence in sheep varied with the region, age and the serological methods from 6.9 to 71% (Dubey et al., 2014a). In the UK, of the 3539 sera collected from 227 sheep flocks, 2619 (74%) were found to be positive for *T. gondii* specific antibodies (Hutchinson et al., 2011). In France, applying a low cut off titre of 1:≥6 in MAT the overall estimate of the *T. gondii* seroprevalence was 17.7% (11.6–31.5%) for lambs and 89% (73.5–100%) for adult sheep (Halos et al., 2010). In a Scandinavian country (Norway), 55 of 73 flocks (75%) had one or more serologically positive animals, while 377 of 2188 (17%) of the individual samples tested positive for *T. gondii* antibodies (Stormoen et al., 2012).

In Oceania, 1917 out of 2254 (85%) sheep sera tested in New Zealand were positive, using a titre of 1:≥16, and 1384/2254 (61%) with a titre of 1:≥64 using a latex agglutination test. All 198 ewe flocks tested were seropositive for antibodies to *T. gondii*, at a titre of 1:≥16, and all but three were at a titre of 1:≥64 (Dempster et al., 2011).

Isolation of viable parasites from tissues of small ruminants corroborate serological findings and confirm that these species are important intermediate hosts. In sheep, viable *T. gondii* has been detected in brain, heart, diaphragm and different muscles (Dubey, 2010b; Opsteegh et al., 2016). Due to the fact that *T. gondii* readily disseminates into the edible tissues of sheep, this parasite represents a risk for consumers (Belluco et al., 2016; Opsteegh et al., 2016). In goats, brain and heart also rank high on the list of predilection organs and muscle tissues had high within study scores, and ranked first when combined in the meat/muscle category (Opsteegh et al., 2016). These results are corroborated by studies in different areas of the world. For instance, the proportion sheep carcasses in France carrying live parasites according to bioassay results was estimated at 5.4% (3–7.5%) (Halos et al., 2010). In the US, 53 isolates of *T. gondii* were obtained from 68 seropositive lambs sampled at the slaughterhouse (Dubey et al., 2008). In another study in this country, hearts of goats obtained from a local grocery store were examined for *T. gondii* infection and the parasite was isolated from 29 out of 112 animals (Dubey et al., 2011).

#### 2.2.2 Possible routes of infection in sheep and goats

Horizontal transmission of *T. gondii* to small ruminants by the oral uptake of environmentally resistant oocysts through contaminated fodder or water is considered the most important route of transmission (Buxton and Losson, 2007; Dubey, 2010b). It is generally assumed that <2% of sheep become infected congenitally and <4% of the persistently infected sheep transmit the infection to their offspring (reviewed in Dubey (2010b) and Innes et al. (2009)). Recrudescence of a chronic infection and the endogenous trans-placental transmission of the parasite to offspring was described in goats (Dubey, 1982). In addition, it was proposed some years ago that a repeated transplacental transmission in sheep was more common than previously thought (Williams et al., 2005) and recent descriptions from Brazil seem to corroborate this hypothesis (Dos Santos et al., 2016; Klauck et al., 2016). Further studies are needed to assess the possibility that certain breeds are more susceptible to endogenous vertical transmission in chronically infected ewes or that vertical transmission is a trait of particular *T. gondii* strains or genotypes.

Possible alternative routes are venereal or galactogenic transmission. Several studies have identified *T. gondii* DNA in semen samples from rams and male goats, either from natural cases of infection (Bezerra et al., 2014) or from animals experimentally inoculated (W.D. Lopes et al., 2013; Santana et al., 2010). Furthermore, the transmission of the infection to sheep and goats through semen has also been proven, both under mating with experimentally infected rams (W.D. Lopes et al., 2013) or through artificial insemination with semen spiked with *T. gondii* tachyzoites (Consalter et al., 2017; Wanderley et al., 2013). On the other hand, the epidemiological significance of this route might be limited (Buxton, 1998). Similarly, milk may also pose a risk of infection to lambs or goat kids, as *T. gondii* DNA has been identified in milk samples from naturally infected ewes and goats (de Santana Rocha et al., 2015; Saad et al., 2018), and bioassay results in raw milk suggest its infective potential (Chiari and Neves, 1984; Dehkordi et al., 2013). However, it needs to be mentioned that the latter findings have been challenged and their epidemiological significance has been questioned (Dubey and Jones, 2014). Even if these alternative routes of transmission are possible in small ruminants, it still needs to be established, to which extent they contribute to infection.

#### 2.2.3 Disease caused by *T. gondii* in naturally infected sheep and goats

It has been estimated that toxoplasmosis is responsible of 10 to 23% of ovine abortions in Europe or USA (Dubey, 2009b). Recent reports have shown that also in other regions of the world, as in the Middle East and South America, *T. gondii* infections are associated with 3 to 54% of ovine abortion (Table 1).

**Table 1:** Reports of *Toxoplasma gondii* induced abortions in small ruminants since 2010.

Country	No. of placentas, fetuses and stillborn lambs examined (sheep/goats)	No. farms tested (sheep/goats)	No. of submissions (sheep/goats)	% positive, total or ovine/caprine	Diagnostic methods				Observations	Reference
					Other causes investigated	IHC	PCR	Fetal serology		
Brazil	35	n.a.	n.a.	14.3	No	No	Yes	No	Ovine abortions	de Moraes et al. (2011)
Great Britain	n.a.^a^	n.a.	n.a.	23.7	Yes^b^	n.a.^a^	Yes^b^	Yes^b^	Ovine abortions	Carson and Group (2017)
Iran	325	n.a.	n.a.	5	No	No	No	Yes	Ovine abortions	Razmi et al. (2010)
	18	n.a.	n.a.	66	No	No	Yes	No	Ovine abortions	Habibi et al. (2012)
	200	n.a.	n.a.	13.5	No	No	Yes	No	Ovine abortions	Rassouli et al. (2011)
	37	n.a.	n.a.	54	No	No	Yes	Yes	Ovine abortions	Danehchin et al. (2016)
Ireland		66	n.a.	17	Yes	Yes	Yes	Yes	Ovine abortions	Gutierrez et al. (2012)
Jordan	106 (66/40)	n.a.	n.a.	31	No	No	Yes	No	Ovine/caprine abortions	Abu-Dalbouh et al. (2012)
Netherlands	n.a.	n.a.	452 (282/170)	10.6/5.9	Yes	Yes	No	No	Ovine/caprine abortions	van den Brom et al. (2012)
	n.a.	n.a.	81 (57/24)	16.7/14.0	Yes	Yes	No	No	Ovine/caprine abortions	van Engelen et al. (2014)
Spain	100 (74/26)	n.a.	n.a.	5.4/3	Yes	No	Yes	No	Ovine/caprine abortions	Moreno et al. (2012)
Switzerland	30	n.a.	n.a.	10	Yes	No	Yes	No	Ovine/caprine abortions	Schnydrig et al. (2017)

a n.a. = not applicable.

b Based on https://veterinaryrecord.bmj.com/content/vetrec/183/17/528.full.pdf (last accessed 2019-01-22).

The only evident clinical sign associated with acquired toxoplasmosis (horizontal transmission), is a brief episode of fever and lack of appetite from about 3–6 days after infection and lasting usually for about 4–5 days, but sometimes also for up to 10 days (Buxton et al., 1982; Buxton et al., 1988; Castano et al., 2016; Dubey, 1981; Esteban-Redondo et al., 1999; McColgan et al., 1988). In contrast, congenital transmission has severe consequences for the fetus. Whether the trans-placental transmission causes the death of the fetus partly depends on the time of gestation when infection occurs. If the dam was infected in early gestation, at a time when the immune system of the fetus is still immature, vertical transmission commonly results in fetal death and resorption. However, when infection occurs at mid gestation, abortion or the delivery of a stillborn lamb are the most common outcomes, while dams infected late in gestation may deliver a stillborn or congenitally infected weak or clinically normal offspring (Buxton and Losson, 2007). Macroscopic lesions in cases of abortion are restricted to the cotyledons, where multifocal areas of necrosis, macroscopically visible as white foci of variable size are suggestive for toxoplasmosis (Buxton and Finlayson, 1986; Buxton et al., 1982). Microscopically, multifocal necrosis, commonly associated with the infiltration of non-purulent lymphoid cells, could be found in placentomes or fetal organs, mainly brain, liver or lung (Buxton and Finlayson, 1986).

#### 2.2.4 Effects of experimental infections in sheep and goats

The precise mechanisms responsible for *T. gondii* abortion in small ruminants are not yet fully understood. The most recent studies, employing the oral route for administering oocysts as infective parasitic stage are summarized in Table 2. The outcome of experimental infections might be affected by the viability of oocysts which needs to be confirmed prior to use (Dubey, 2010b). In addition, the *T. gondii* strain characteristics including the virulence, which seems to change after repeated passages (Saraf et al., 2017), need to be taken into account. Results of experimental infections have clearly shown that the gestational age, in particular the stage of maturation of the fetal immune system has an important effect on the pathogenesis (Buxton and Finlayson, 1986). In addition, the cellular immune response of the dam, mainly mediated by IFN-γ, is of importance in controlling the parasite multiplication (Innes et al., 1995). The experimental inoculation of sheep and goats has also helped to demonstrate that toxoplasmosis in small ruminants could also cause early abortion shortly after infection. In these early abortions, invasion and multiplication of the parasite in the placenta or fetus could not be demonstrated (Owen et al., 1998; Rosa et al., 2001). Although the cause of these early abortions was thought to be high fever or hormonal dysregulation (Dubey, 2010b), recent studies have shown that they are related to vascular lesions in the placenta and leukomalacia in the fetal brain (Castano et al., 2014). All together, these results suggest that the pathogenesis of early abortion is different from the classically described, which is based on the multiplication of the parasite and subsequent lesions in the placenta and target fetal organs. The mechanisms underlying the early abortions in this disease are still unknown. Bearing these observations in mind, there are still several gaps in the knowledge of small ruminant toxoplasmosis that warrant further characterization of the experimental models for ovine and caprine toxoplasmosis and investigation on how different variables, e.g. *T. gondii* strain or isolate virulence, previous immunization or individual susceptibility could affect the pathogenesis of this disease.

**Table 2:** Experimental studies in sheep orally inoculated with *Toxoplasma gondii* oocysts or tissue cysts. Studies published since 2010.

Designation of *Toxoplasma gondii* isolate	Dose	Numbers and age category	Remarks	Reference
M4	3000	28 sheep	Inoculated with sporulated oocysts at day 90 of gestation	Gutierrez et al. (2010)
	500,000; 5000	16 lambs	Inoculated with sporulated oocysts.	Benavides et al. (2011)
	3000	9 sheep	Inoculated with sporulated oocysts at day 90 of gestation	O'Donovan et al. (2012)
	3000	15 sheep	Inoculated with sporulated oocysts at day 90 of gestation	Marques et al. (2012)
	2000; 500	24; 24 sheep	Inoculated with sporulated oocysts at day 90 of gestation (n = 24) and at day 120 (n = 24)	Castano et al. (2014)
	500	33 lambs	Inoculated with sporulated oocysts	Katzer et al. (2014)
	50	27 sheep	Inoculated with sporulated oocysts at three terms of gestation	Castano et al. (2016)
PRU	400; 400; 100	36; 54; 33 sheep	Inoculated with sporulated oocysts at mid-gestation	Mevelec et al. (2010)
	3000	13 lambs	Inoculated with tissue cysts	Verhelst et al. (2014)
	3000	4 sheep	Inoculated with tissue cysts	Verhelst et al. (2015)
P	200,000	4 rams	Inoculated with sporulated oocysts.	Lopes et al. (2011)
ME49; VEG	2500; 2500	20 sheep	Inoculated with sporulated oocysts at three terms of gestation of chronically infected ewes.	Dos Santos et al. (2016)
ME49	500; 50; 10	5; 5; 5 sheep	Inoculated with sporulated oocysts at day 90 of gestation	Sánchez-Sánchez et al. (2019)
TgShSp1	500; 50; 10	6; 6; 6 sheep	Inoculated with sporulated oocysts at day 90 of gestation	Sánchez-Sánchez et al. (2019)

### 2.3 Chickens and other poultry

#### 2.3.1 Prevalence in chickens and other poultry

*Toxoplasma gondii* infection in free-ranging poultry is an indicator of environmental contamination with *T. gondii* oocysts (Dubey, 2010b). Strains isolated from local poultry probably mirror the *T. gondii* genotypes prevailing in a region (Dubey, 2010a, Dubey, 2010b). The prevalence of *T. gondii* infections in poultry depends on a number of factors. The type of husbandry seems to be very important. In poultry originating from free-range or small backyard farms, the *T. gondii* prevalence is usually higher than in poultry kept indoors (Guo et al., 2015; Maksimov et al., 2011; Schares et al., 2017a; Yang et al., 2012).

In chickens, there is a number of recent articles summarizing the seroprevalence of antibodies to *T. gondii* (Bangoura et al., 2011; Deng et al., 2018; Dubey, 2010a; Guo et al., 2015; Shokri et al., 2017). Prevalence estimates are often not comparable among studies because different serological tests have been applied and sampled farms may differ for example in farm type and size, feed source, presence or absence of cats, rodent or bird control and water quality (Bangoura et al., 2011; Dubey, 2010a; Schares et al., 2017a). In some studies, a low specificity of the serological tests may have overestimated the seroprevalence or a low sensitivity may have led to an underestimation. Overall, the *T. gondii* seroprevalences in chickens ranged between 0 and 100% (Ayinmode and Olaosebikan, 2014; Bangoura et al., 2011; Deng et al., 2018; Dubey, 2010a; Matsuo et al., 2014).

Only few studies on *T. gondii* prevalence in turkeys have been published. Apparent seroprevalence varies largely between studies and ranges from 0% (Czech Republic), 11% (Brazil), 20% (Germany) to 59% (Egypt) (Bangoura et al., 2011; El-Massry et al., 2000; Harfoush and Tahoon Ael, 2010; Koethe et al., 2011; Sa et al., 2016).

The *T. gondii* seroprevalence in ducks and geese, as summarized for Poland, Czech Republic, Germany and Norway, varied between 1.7 and 21% in ducks and between 5.9 and 43% in geese (reviewed by Bangoura et al. (2011)). Only 3.5% of geese in Kazakhstan were seropositive (Bangoura et al., 2011). *T. gondii* seroprevalences reported for China in ducks and geese were in the range of 6.3–13.9% (Cong et al., 2012; Yang et al., 2012). The highest seroprevalences in ducks were reported from Egypt (50–55%) (El-Massry et al., 2000; Harfoush and Tahoon Ael, 2010) and Malaysia (30%) (Puvanesuaran et al., 2013).

#### 2.3.2 Possible routes of infection in chickens and other poultry

Due to the ground feeding behavior of poultry, the oral ingestion of material or water contaminated with *T. gondii* oocysts is most likely the main route of infection (Dubey, 2010b). Water may be contaminated with *T. gondii* oocysts (Isaac-Renton et al., 1998). Thus, oocyst contaminations of water can be of particular importance as a source of infection for waterfowl. Infected rodents and other intermediate hosts on farms may serve as a reservoir (Schares et al., 2017a). Poultry such as chickens, turkeys, ducks and geese are omnivorous, i.e. they also may feed on earthworms, cockroaches and other insects, which may harbor, or could be contaminated with oocysts (Bettiol et al., 2000; Ruiz and Frenkel, 1980; Wallace, 1971, Wallace, 1972). In addition, poultry may predate on small rodents as putative carriers of *T. gondii* tissue cysts. In an experimental setting, turkeys became infected after inoculation with brains of chronically infected mice (Koethe et al., 2015) and also chickens fed tissue cysts became infected (summarized by Dubey (2010a)). There is, however, lack of information, to which extent such different routes of infection (i.e. infections via tissue cysts) are relevant under natural conditions. Vertical transmission of *T. gondii* in poultry has been discussed in the past, but extensive experiments in chickens indicated that this route of infection can be left outside the focus (Biancifiori et al., 1986; Boch et al., 1966).

#### 2.3.3 Disease caused by *T. gondii* in naturally infected poultry

In general, chickens, turkeys, ducks and geese rarely develop clinical signs after infection with *T. gondii* (Dubey, 2010a). Worldwide, there are only few reports on clinical toxoplasmosis in naturally infected poultry (Dubey, 2010a, Dubey, 2010b). It has to be kept in mind, however, that some of the clinical cases reported as caused by *T. gondii* may have been triggered by other infections (e.g. viral) or complicated by other diseases (Dubey, 2010a).

No *T. gondii* genotype-dependent virulence in adult chickens has been recorded and even South-American strains, highly virulent to mice, seem to be avirulent in chickens (Hamilton et al., 2017; Vaudaux et al., 2010). However, it has to be mentioned here that young chickens (1-month-old), infected by oocysts of *T. gondii* Type I (GT1 strain) developed clinical toxoplasmosis, whereas those infected by oocysts of *T. gondii* Type II (ME49) did not develop any clinical signs (Dubey et al., 1993b). Chicken-line dependent differences in mortality after experimental inoculation of chicks with recombinant *T. gondii* clones suggested that, in addition to the parasites genotype, also genetic factors of the host may play an important role in the development of clinical toxoplasmosis in chickens (Schares et al., 2017b). Age of the chicken seems to be a very because also in another study, 10-day old chickens showed mortality in a dose-dependent fashion (S. Wang et al., 2015).

Reports of toxoplasmosis in magpie (*Anseranas semipalmata*) and Hawaiian geese (*Branta sandvicensis*) (Dubey et al., 2001; Work et al., 2015) suggest that there might be differences in susceptibility for *T. gondii* infection and toxoplasmosis between different species of Anseriformes. By contrast, we could not find reports on clinical toxoplasmosis in domestic geese (*Anser anser*).

#### 2.3.4 Effects of experimental infections in livestock poultry

The susceptibility of poultry to experimentally induced toxoplasmosis depends on the infectious dose, the parasite strain, stage, the route of infection and, as mentioned above, the age of the animal (Dubey et al., 1993b; Schares et al., 2017b). In chickens, parenteral infection with *T. gondii* tachyzoites or oral infection with oocysts rarely cause clinical signs (Dubey, 2010a). However, in the case of intracranial infections using encysted *T. gondii*, the animals developed severe cerebral toxoplasmosis (Bickford and Saunders, 1966; Dubey, 2010a).

No clinical toxoplasmosis was reported in turkeys either after experimental oral infection with different doses of *T. gondii* oocysts (Bangoura et al., 2013; Dubey, 2010b; Dubey et al., 1993a), or intravenous inoculation with varying doses of tachyzoites (with strains representative for *T. gondii* clonal Types I, II and III) (Maksimov et al., 2018; Zöller et al., 2013).

The results of experimental infections in ducks and geese with oocysts (Bartova et al., 2004; Maksimov et al., 2011) and intravenous infections with tachyzoites (Maksimov et al., 2011) showed that also these animal species were resistant against clinical toxoplasmosis regardless of the parasite stage used for infection.

### 2.4 Cattle

#### 2.4.1 Prevalence in cattle

Seroprevalence estimates in cattle, if obtained by highly specific tests, can be of value to monitor the exposure of cattle to *T. gondii*. However, these serological data must be interpreted with care as studies conducted with bioassay experiments suggest that in the vast majority of seropositive cattle there was no evidence for the presence of viable *T. gondii* infections. This has been also shown by analyses of naturally exposed animals, in some studies with very large populations of cattle (Boch et al., 1965a; Dubey et al., 2005; Dubey and Streitel, 1976; Fortier et al., 1990; Freyre et al., 1990; Jacobs and Moyle, 1963; Passos et al., 1984). There are only a few reports on naturally exposed cattle, in which positive *T. gondii* bioassays indicated viable infections (Arias et al., 1994; Catar et al., 1969; de Macedo et al., 2012; Dubey, 1992; Jacobs et al., 1960). This is in a sharp contrast to the findings in small ruminants as discussed above.

With the advent of new methodologies, i.e. genome detection by PCR, a number of studies utilizing these techniques yielded very high proportions (up to 10 or 20%) of *T. gondii* genome positive samples in cattle tissues (Amdouni et al., 2017; Azizi et al., 2014; Berger-Schoch et al., 2011; Campo-Portacio et al., 2014; Ergin et al., 2009; Hosein et al., 2016; Mahami-Oskouei et al., 2017; Opsteegh et al., 2011; Rahdar et al., 2012; Wyss et al., 2000). Keeping in mind the failure of many large-scale studies to find viable *T. gondii* in bovine tissues, the validity of these reports on the detection of *T. gondii* genome fragments has to be questioned. Detection of genome fragments of *T. gondii* in absence of positive bioassays should not be regarded as conclusive.

A recent meta-analysis revealed the possibility of geographic differences in the proportion of *T. gondii*-positive cattle. A significantly higher proportion of positive cattle was found in Central America as compared to North America (Belluco et al., 2016). This may indicate that the susceptibility of cattle to *T. gondii* is influenced by the genotype of the parasite, which largely varies in different regions of the world (Bertranpetit et al., 2017; Chaichan et al., 2017; Shwab et al., 2014). However, these considerations are hypothetical and need to be addressed in future studies. In addition, differences in husbandry conditions, hygienic situations, in climate and in other factors may affect the extent, to which cattle from different regions are exposed to *T. gondii*. Therefore, there is a need to confirm the detection of *T. gondii* genome positive samples in cattle by additional experiments, thus assessing the presence of viable parasites.

Over the past decades, numerous articles have been published on the seroprevalence of *T. gondii*-specific antibodies in taurine cattle. Many of these publications have been reviewed before, with a global scope (Dubey, 1986a, Dubey, 2010b; Tenter et al., 2000) or, more recently, by focusing on the situation in particular regions of the world like China (Deng et al., 2018), South Asia (Khan et al., 2017) and Africa (Tonouhewa et al., 2017). Overall, theses summaries show a large variation in the reported proportions of positive findings and the summarizing estimates were 9.5% for cattle in China (Deng et al., 2018), 27.9% in South Asia (Khan et al., 2017) or 12% in Africa (Tonouhewa et al., 2017).

#### 2.4.2 Possible routes of infection in cattle

It is generally accepted that most cattle become infected orally, through ingestion of feed or water contaminated with *T. gondii* oocysts. Many experimental infections in cattle, especially earlier ones, used oocysts as the inoculum, thus demonstrating that cattle are susceptible to this infective stage (Burrells et al., 2018; Costa et al., 2011; de Oliveira et al., 2001; Dubey and Thulliez, 1993; Esteban-Redondo et al., 1999; Stalheim et al., 1980). However, usually large numbers of oocysts were administered, but we are not aware of any experiments that aimed at establishing the minimum infective dose for cattle.

There are also reports on bovine infections with viable *T. gondii* caused by oral inoculation with *T. gondii* tissue cysts (10^3^) (Costa et al., 2011) or brains of chronically infected mice (Rommel et al., 1966). Although cattle are herbivores, infections through accidental ingestion of tissue cysts may occur, i.e. if cattle feed is contaminated with fresh tissue of an infected intermediate host.

In infection with *Neospora caninum*, an apicomplexan parasite closely related to *T. gondii*, vertical transmission after acute or chronic infection is of utmost importance in cattle (Dubey et al., 2017). However, for *T. gondii* the situation seems to be different. Although there are reports on the detection of *T. gondii* genome fragments in aborted bovine fetuses (Ellis, 1998; Gottstein et al., 1998), the isolation of viable *T. gondii* parasites from bovine fetuses was achieved only occasionally (Canada et al., 2002; Costa et al., 1977) or not at all (Conrad et al., 1993). Experimental inoculations with tachyzoites resulted in abortion or vertical transmission (Stalheim et al., 1980; Wiengcharoen et al., 2011), but the epidemiological significance of these findings is not clear, because the presence of *T. gondii* in the aborted fetuses was not confirmed. Overall, if vertical transmission of *T. gondii* naturally occurs in cattle, it seems to be a rare event. However, the large genetic variability between *T. gondii* populations worldwide should be kept in mind, which may result in a variety of biological traits that may also include differences in virulence in cattle. In the light of this variation, findings in North America and Europe with isolates prevailing in these regions should not be generalized without confirmation.

#### 2.4.3 Disease caused by *T. gondii* infections in cattle

Reports on clinical toxoplasmosis in naturally infected cattle are rare. This suggests that cattle are resistant to infection and to clinical toxoplasmosis. Although clinical signs and histological alterations were recorded after experimental infection, natural cases of clinical toxoplasmosis in cattle comprised only of abortions in association with the isolation of *T. gondii* from the aborted fetuses (Canada et al., 2002). It is not clear, however, whether the infection with *T. gondii* had caused the abortions.

#### 2.4.4 Effects of experimental infections in cattle

After experimental infection, febrile reactions starting 2 days post inoculation at the earliest and lasting up to 15 days p.i. have been regularly reported (Burrells et al., 2018; Costa et al., 1977; Esteban-Redondo et al., 1999; Munday, 1978; Rommel et al., 1966; Stalheim et al., 1980; Wiengcharoen et al., 2011). Bovine infection with *T. gondii* regularly leads to a parasitemia, which seems to be responsible for the elevated temperatures observed in inoculated animals shortly after infection (de Oliveira et al., 2001; Stalheim et al., 1980). In one study, the parasitemia was even recorded up to 62 days p.i. (Costa et al., 1977). In addition, respiratory distress, nasal discharge, and hyperemia of the conjunctivae were reported in the latter study (Costa et al., 1977).

Reports on mortality in inoculated animals are rare. It occurred in cases of calves inoculated with oocysts or intravenously with tachyzoites, but only in the latter the infection was confirmed (Stalheim et al., 1980). In another experiment, two out of four dams inoculated with *T. gondii* tachyzoites became recumbent and were euthanized (Wiengcharoen et al., 2011). In this case, adult cattle were affected a long time after inoculation (2 to 3 month p.i.) and this finding represented a surprising exception among a series of experiments, where inoculated cattle developed no or only mild clinical signs (Beverley et al., 1977; Burrells et al., 2018; Costa et al., 1977; Dubey, 1983; Esteban-Redondo et al., 1999; Munday, 1978; Rommel et al., 1966).

### 2.5 Horses and other equids

#### 2.5.1 Prevalence in horses and other equids

A relatively large number of studies report on the seroprevalence of antibodies against *T. gondii* in horses, mules and donkeys world-wide. Most but not all of the publications have been reviewed previously (Dubey, 2010b; Dubey et al., 2014b; Tassi, 2007; Tenter et al., 2000). The study results are difficult to compare because different, not always validated serological methods and various cut-offs have been applied. In addition, the equids selected for the studies differed largely in number, age, origin and the purpose for keeping the animals. Currently, there is no reference standard available to validate serological tests in horses properly. A recent attempt to correlate serological test results (i.e. results by MAT and a commercially available ELISA) with those of *T. gondii* PCR on horse meat samples largely failed (Aroussi et al., 2015). There was almost no correlation between the serological data and *T. gondii* genome detection using a highly sensitive magnetic capture PCR (Aroussi et al., 2015). Nevertheless, there is no doubt that horses can harbor viable *T. gondii*, which could be isolated from tissues of both, naturally (Evers et al., 2013; Gennari et al., 2015; Klun et al., 2017; Shaapan and Ghazy, 2007) or experimentally infected animals (Al-Khalidi et al., 1980; Dubey, 1985). The results indicated that viable *T. gondii* can persist in edible tissues up to 476 days after infection (Dubey, 1985). In addition, imported meat from infected horses was suspected as cause of toxoplasmosis in France (Elbez-Rubinstein et al., 2009; Pomares et al., 2011). A recent example on isolation of viable *T. gondii* from horse shows that truly infected horse may remain seronegative or develop only a low specific antibody titre in particular serological tests such as the MAT (Klun et al., 2017). Currently, serological responses in equids do not seem to be reliable indicators for viable infections; this is similar to the situation in cattle. Nevertheless, positive antibody responses indicate the exposure of equids to *T. gondii* and could thus be used to identify risk factors for their exposure to *T. gondii*. Reported seroprevalence for equids range in South America from 3 to 90% (Cazarotto et al., 2016; Costa et al., 2012; Dangoudoubiyam et al., 2011; de Oliveira et al., 2013; Dubey, 2010b; Evers et al., 2013; Finger et al., 2013; Gennari et al., 2015; Portella et al., 2017; Ribeiro et al., 2016; Tassi, 2007; Venturi et al., 2017), in North America from 0 to 73% (Alvarado-Esquivel et al., 2015; Alvarado-Esquivel et al., 2012b; Dubey, 2010b; Dubey et al., 2014b; James et al., 2017; Schale et al., 2018; Tassi, 2007), in Europe from 0 to 55% (Bartova et al., 2015; Dubey, 2010b; Garcia-Bocanegra et al., 2012; Karatepe et al., 2010; Klun et al., 2017; Kouam et al., 2010; Machacova et al., 2014; Mancianti et al., 2014; Papini et al., 2015; Pastiu et al., 2015; Tassi, 2007; Zhou et al., 2017), in Asia from 0 to 71% (Aharonson-Raz et al., 2015; Alanazi and Alyousif, 2011; Dubey, 2010b; Hajialilo et al., 2010; Lee et al., 2014; Mancianti et al., 2014; Masatani et al., 2016; Matsuo et al., 2014; Miao et al., 2013; Raeghi et al., 2011; Razmi et al., 2016; Saqib et al., 2015; Tassi, 2007; Tavalla et al., 2015; J.L. Wang et al., 2015; Yang et al., 2013), in Africa from 14 to 45% (Ayinmode et al., 2014; Boughattas et al., 2011; Haridy et al., 2010) and 2% in Australia (Tassi, 2007).

#### 2.5.2 Possible routes of infection in horses and other equids

In the case of equids, oral infection by oocysts is the most probable route as it has been confirmed by a number experimental infections using different doses of oocysts (Al-Khalidi et al., 1980; Altan et al., 1977; Dubey, 1985; Marques et al., 1995), i.e. doses of 10^5^ (Al-Khalidi et al., 1980), 10^4^ (Dubey, 1985), 10^6^ (Altan et al., 1977), or up to 1.5 × 10^5^ (Marques et al., 1995).

Rodents are intermediate hosts of *T. gondii* and regarded as a source of infection especially in omnivorous animals like pigs. Since it has been demonstrated that a large proportion of horses would eat meat and may become infected by *Trichinella spiralis* via this route (Murrell et al., 2004), it is tempting to speculate that the oral ingestion of carcasses of *T. gondii* infected rodents or other small intermediate hosts may represent another potential source of infection for equids.

Reports on transplacental *T. gondii* transmission in experimentally infected mares (Marques et al., 1995) have to be interpreted carefully and need further investigation because infections with other related parasites like *Sarcocystis neurona* or *N. caninum* need to be ruled out.

#### 2.5.3 Disease caused by *T. gondii* infections in horses and other equids

The general view is that toxoplasmosis, i.e. disease caused by *T. gondii* infection, is rather rare in equids, after both natural and experimental infection (Dubey, 2010b; Tassi, 2007). *T. gondii* DNA has been detected in the eyes of an aborted foal (Turner and Savva, 1992) and in the placenta of a mare that foaled normally (Turner and Savva, 1990). Together with the transplacental transmission reported in an experimental study, these results may indicate that *T. gondii* could be occasionally involved in equine abortion (Marques et al., 1995), but further studies are necessary to clarify this issue. It was recently discussed if *T. gondii* is involved in equine protozoal myeloencephalitis (EPM) (James et al., 2017; Schale et al., 2018). A case control study conducted in California found an association between high levels of *T. gondii* IFAT titres (1:≥160 or 1:≥320) and the presence of neurologic signs compatible with EPM (James et al., 2017). Another study, not designed as a case-control study but thoroughly assessing EPM cases, revealed only low proportions of *T. gondii* (and also *Neospora* sp.) positive animals in this group of patients, contradicting an involvement of *T. gondii* in EPM. In this study, *S. neurona* was identified as the most probable cause of EPM.

An earlier study conducted in the UK reported on the presence of *T. gondii* DNA in the eye of a pony (Turner and Savva, 1991). The significance of this finding and the involvement of *T. gondii* as a possible cause of blindness in horse are unknown. However, after experimental infection of a pony with *T. gondii*, the infection was also observed in the eye (Dubey, 1985).

#### 2.5.4 Effects of experimental infections in horses

In equids experimentally inoculated with large numbers of oocysts (10^4^), mild fever was observed in few animals, while the others remained clinically normal (Dubey, 1985; Dubey and Desmonts, 1987). In ponies orally inoculated with a high number of oocysts and in addition immunosuppressed by corticosteroid, 8 out of 9 ponies developed fever between days 2 and 15 p.i. (Al-Khalidi et al., 1980). In an earlier study, ponies, not immunosuppressed but orally inoculated by 10^6^ oocysts did not develop clinical signs (Altan et al., 1977). Horses inoculated intravenously with tachyzoites (i.e. 3.28 × 10^7^ or 2.19 × 10^7^, strain RH) developed fever between 4 to 8 days after inoculation and ocular discharge from 10 to 26 days post inoculation (Sposito Filha et al., 1992).

## 3 Potential risk factors for infection in livestock

Raw and undercooked meat are regarded as one of the main sources of *T. gondii* infections for humans. Knowledge on risk factors for the infection with *T. gondii* in livestock and an assessment of the importance of these risk factors is essential to ensure safe food and intervene effectively. This section is partially based on previous reviews (Guo et al., 2015; Opsteegh et al., 2016) and was extended including the most recent reports in the field. We present a brief overview on the existing literature, but no deeper meta-analysis. We chose to categorize the literature data on individual risk factors only into “statistically significant” and “not statistically significant”, not discriminating between the statistical testing methods used. Mainly reports were included, in which risk analysis was based on the seropositivity of livestock animals. In addition, the review was restricted to reports on the main livestock species, i.e. pigs, small ruminants (sheep and goats), cattle, equids (horses, donkey and mules) and poultry (chickens, ducks and geese).

**Fig. 1:**
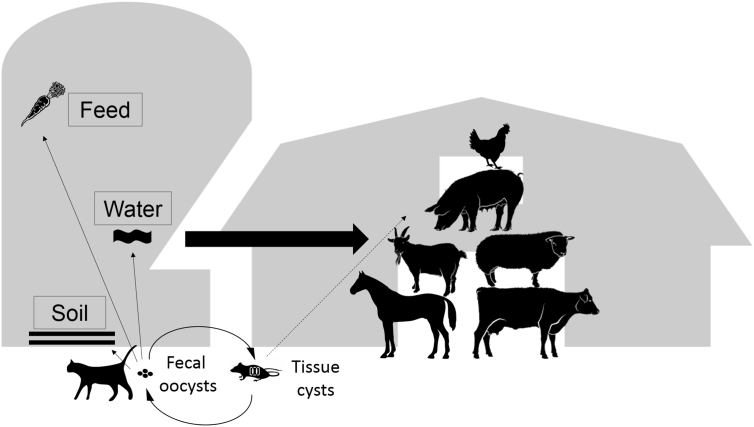
*Toxoplasma gondii* life cycle highlighting conditions of horizontal transmission concerning livestock infection.

To identify specific risk factors for infection, it is crucial to know the most important routes, by which livestock can acquire the infection. These routes include oocyst contaminations of feed, water or the environment, and the ingestion of tissues of infected intermediate hosts like rodents (Fig. 1).

The risk of infection and particularly of infection routes on individual farms (Fig. 1) are influenced by several indirectly acting factors. They include factors related to particular rearing systems of specific livestock groups. There are no general rules to define or to assess such risk factors, which makes a comparison of the results between studies difficult or even impossible. Although the spectrum of risk factors reported in the literature is very heterogenic, we tried to categorize them as much as possible to come to some more general conclusions. In our analysis, we looked for the basic, overarching factors and stratified the records by livestock species. If both, univariable and multivariable statistical analyses had been reported for a specific factor, we included only the results of multivariable statistical analyses, i.e. the results of analyses, in which the specific factor had been examined together with at least one other factor. Because almost all of the studies included were retrospective studies it has to be kept in mind that associations or statistical effects of factors identified by this type of studies are not entirely conclusive and only allow the generation of hypotheses.

### 3.1 General factors

#### 3.1.1 Age

The association of age with the risk of a *T. gondii* infection has been examined in a number of studies (Table 3). Generally, the wide spread of *T. gondii* and the extremely broad host spectrum of the parasite leads to a time of exposure to the infective stages of the parasite that is proportional to the age of the animal. The age of the animals often depends on the production type, i.e. meat-producing animals are usually slaughtered at younger age, whereas animals reared for dairy production, reproduction or recreational purposes often live much longer. Overall, literature data confirm the expected association with age, i.e. in most studies age appeared as a risk factor for infection with *T. gondii* (Table 3). Due to its general importance, the factor “age” should be always included as one of the explanatory variables, if risk factor analyses on postnatal infection among animals with varying age are performed. Age is prone to act as a confounder or effect modifier in statistical analyses (Schares et al., 2017a).

**Table 3:** The effect of age on the seropositivity for *T. gondii* in livestock species.

Factor	Species	Statistically significant	Not statistically significant
Higher age	Pigs	17, 20, 25, 34, 33, 39, 53, 57 (p), 75, 80, 86, 94	6, 28, 73, 96
	Sheep	13, 15, 23, 35, 36, 41, 42, 45, 46, 50, 57, 70, 85, 88, 90	7, 68, 72, 92, 95, 98
	Goats	3, 8, 19 (p), 24, 30, 35, 47, 57, 68, 88	92, 95
	Cattle	–	79, 95, 99
	Equids	102, 104	5, 11, 31, 40, 54, 100, 101, 103
	Chicken	4, 93	–
Finishing period	Pigs	75	–
Age below 12 months	Sheep	16 (p)	–
Younger age	Cattle	38, 79	53
Age <24 months	Cattle	48 (p)	–
Age >24 months–96 months + >120 months (dairy and mixed dairy)	Cattle	48	–
Age >48 months–72 months (beef and mixed beef)	Cattle	48	–

(p) = protective factor; coding of references: 3 = Alvarado-Esquivel et al. (2011), 4 = Alvarado-Esquivel et al. (2012a), 5 = Alvarado-Esquivel et al. (2012b), 6 = Alvarado-Esquivel et al. (2014), 7 = Alvarado-Esquivel et al. (2013a), 8 = Alvarado-Esquivel et al. (2013b), 11 = Cazarotto et al. (2016), 13 = Andrade et al. (2013), 15 = Cosendey-KezenLeite et al. (2014), 16 = D'Alencar Mendonca et al. (2013), 17 = Damriyasa et al. (2004), 19 = de Moura et al. (2016), 20 = de Sousa et al. (2014), 23 = Deksne et al. (2017), 24 = Deng et al. (2016), 25 = Djokic et al. (2016), 28 = Esteves et al. (2014), 30 = Garcia et al. (2012), 31 = Garcia-Bocanegra et al. (2012), 33 = Garcia-Bocanegra et al. (2010a), 34 = Garcia-Bocanegra et al. (2010b), 35 = Gazzonis et al. (2015), 36 = Gebremedhin et al. (2013), 38 = Gilot-Fromont et al. (2009), 39 = Goerlich (2011), 40 = Guerra et al. (2018), 41 = Guimaraes et al. (2013), 42 = Hammond-Aryee et al. (2015), 45 = Holec-Gasior et al. (2015), 46 = Hutchinson et al. (2011), 47 = Iovu et al. (2012), 48 = Jokelainen et al. (2017), 50 = Katzer et al. (2011), 53 = Klun et al. (2006), 54 = Kouam et al. (2010), 57 = A.P. Lopes et al. (2013), 68 = Rego et al. (2016), 70 = Romanelli et al. (2007), 72 = Sakata et al. (2012), 73 = Santoro et al. (2017), 75 = Schulzig and Fehlhaber (2005), 79 = Tan et al. (2015), 80 = Tao et al. (2011), 85 = Vesco et al. (2007), 86 = Villari et al. (2009), 88 = Xu et al. (2015), 90 = Zhang et al. (2016), 92 = Zou et al. (2015), 93 = Schares et al. (2017a), 94 = Samico-Fernandes et al. (2017), 95 = Tilahun et al. (2018), 96 = Bawm et al. (2016), 98 = Yin et al. (2015), 99 = Schoonman et al. (2010), 101 = Bartova et al. (2017), 102 = Machacova et al. (2014), 103 = Miao et al. (2013), 104 = Ribeiro et al. (2016).

#### 3.1.2 Gender

The gender of livestock animals as a putative risk factor has been studied only occasionally (Table 4). Experimental studies in mice and guinea pigs showed a higher susceptibility of females to infection with *T. gondii* (Kittas and Henry, 1979, Kittas and Henry, 1980; Roberts et al., 1995; Roberts et al., 2001). In most of the published studies on livestock recorded, a significant effect for female animals to be serologically positive for *T. gondii* was not detected. Nevertheless, with the exception of two studies, in which males showed an increased risk, females were more frequently seropositive in a few studies conducted with pigs, sheep and goats or equids (Table 4). Whether these apparent associations were in fact related to gender or to other underlying factors, e.g. the way animals of different gender are reared, needs to be questioned. It must be noted that gender frequently shows up as a confounder in epidemiological studies because “gender” may mask these underlying factors (Thrusfield, 2007).

**Table 4:** The effect of female gender on the seropositivity for *T. gondii* in livestock species.

Factor	Species	Statistically significant	Not statistically significant
Female gender	Pigs	73	6, 20, 28, 43, 53, 86, 94
	Sheep	15 (p), 36, 45 (p), 68, 95	7, 23, 35, 41, 59, 72, 81, 88, 90, 92, 98
	Goats	19, 30, 68	8, 35, 81, 88, 92, 96
	Cattle	–	21, 53
	Equids	102, 103	5, 11, 31, 40, 54, 100, 101

(p) = protective factor; coding of references: 5 = Alvarado-Esquivel et al. (2012b), 6 = Alvarado-Esquivel et al. (2014), 7 = Alvarado-Esquivel et al. (2013a), 8 = Alvarado-Esquivel et al. (2013b), 11 = Cazarotto et al. (2016), 15 = Cosendey-KezenLeite et al. (2014), 19 = de Moura et al. (2016), 20 = de Sousa et al. (2014), 21 = de Souza et al. (2016), 23 = Deksne et al. (2017), 28 = Esteves et al. (2014), 30 = Garcia et al. (2012), 31 = Garcia-Bocanegra et al. (2012), 35 = Gazzonis et al. (2015), 36 = Gebremedhin et al. (2013), 40 = Guerra et al. (2018), 41 = Guimaraes et al. (2013), 43 = Herrero et al. (2016), 45 = Holec-Gasior et al. (2015), 53 = Klun et al. (2006), 54 = Kouam et al. (2010), 59 = Magalhaes et al. (2016), 68 = Rego et al. (2016), 72 = Sakata et al. (2012), 73 = Santoro et al. (2017); 81 = Tzanidakis et al. (2012), 86 = Villari et al. (2009), 88 = Xu et al. (2015), 90 = Zhang et al. (2016), 92 = Zou et al. (2015), 94 = Samico-Fernandes et al. (2017), 95 = Tilahun et al. (2018), 96 = Bawm et al. (2016), 98 = Yin et al. (2015), 100 = Almeida et al. (2017), 101 = Bartova et al. (2017), 102 = Machacova et al. (2014), 103 = Miao et al. (2013).

#### 3.1.3 Geographic and regional characteristics

For all species taken into consideration in this review, there were studies reporting on significant differences in seroprevalence with respect to regions or geographic characteristics of the farm locations (Table 5). Many region- and geography-related variables that could possibly affect the survival and presence of *T. gondii* or the exposure of animals to the parasite must therefore be taken into account here. Few studies not only evaluated the differences between certain regions, but also looked into more details concerning the most likely underlying variables, such as mean temperatures, mean rainfall, humidity, altitude or terrain characteristics (Table 5). Most of them found a statistically significant influence of these parameters on the proportion of seropositivity in livestock. Consequently, it is important to take regional differences into consideration but the underlying true effectors such as climatic factors or variables related to differences in animal husbandry need to be included.

**Table 5:** The effect of geographic parameters on the seropositivity for *T. gondii* in livestock species.

Factor	Species	Statistically significant	Not statistically significant
Region, province, municipality, prefecture or district	Pigs	17, 20, 22, 53, 67	6
	Sheep	15, 32, 35, 45, 53, 50, 59, 69, 83, 90, 92, 98, 106	23, 81, 88, 95
	Goats	8, 19, 26, 32, 35, 47, 92	81, 88, 95, 107
	Cattle	32, 48, 53, 95	78, 79
	Equids	40, 54, 101	31
	Chicken	4	89
Altitude	Pigs	6, 86	–
	Sheep	2, 7, 35, 36, 49, 77	14, 81
	Goats	8, 49	35, 81
Mean monthly temperatures	Pigs	34	6
	Sheep	7	–
Mean annual rainfall	Pigs	34	6
	Sheep	7	–
Climate	Pigs	6	–
	Goats	8	–
Relative humidity	Pigs	34	–
Hills relative to plains	Pigs	–	80
Generalized land cover	Sheep	49	–
	Goats	49	–
Distance to next village	Sheep	–	81
	Goats	–	81
Rural environment relative to urban environment	Sheep	–	95
	Goats	–	95
	Cattle	–	95
	Equids	5	–
Terrain waterlogged (versus rough and flat)	Sheep	69	–
Semi-desert	Goats	3	–

Coding of references: 2 = Alvarado-Esquivel et al. (2013a), 3 = Alvarado-Esquivel et al. (2011), 4 = Alvarado-Esquivel et al. (2012a), 5 = Alvarado-Esquivel et al. (2012b), 6 = Alvarado-Esquivel et al. (2014), 7 = Alvarado-Esquivel et al. (2013b), 8 = Alvarado-Esquivel et al. (2013c), 14 = Condoleo et al. (2016), 15 = Cosendey-KezenLeite et al. (2014), 17 = Damriyasa et al. (2004), 19 = de Moura et al. (2016), 20 = de Sousa et al. (2014), 22 = Deksne and Kirjusina (2013), 23 = Deksne et al. (2017), 26 = Djokic et al. (2014), 31 = Garcia-Bocanegra et al. (2012), 32 = Garcia-Bocanegra et al. (2013), 34 = Garcia-Bocanegra et al. (2010b), 35 = Gazzonis et al. (2015), 36 = Gebremedhin et al. (2013), 40 = Guerra et al. (2018), 45 = Holec-Gasior et al. (2015), 47 = Iovu et al. (2012), 48 = Jokelainen et al. (2017), 49 = Kantzoura et al. (2013), 50 = Katzer et al. (2011), 53 = Klun et al. (2006), 54 = Kouam et al. (2010), 59 = Magalhaes et al. (2016), 67 = Pastiu et al. (2013), 69 = Rizzo et al. (2017), 77 = Skjerve et al. (1998), 78 = Sun et al. (2015), 79 = Tan et al. (2015), 80 = Tao et al. (2011), 81 = Tzanidakis et al. (2012), 83 = Verhelst et al. (2014), 86 = Villari et al. (2009), 88 = Xu et al. (2015), 90 = Zhang et al. (2016), 92 = Zou et al. (2015), 95 = Tilahun et al. (2018), 98 = Yin et al. (2015), 101 = Bartova et al. (2017), 106 = Jokelainen et al. (2010), 107 = Shuralev et al. (2018).

#### 3.1.4 Farm management

##### 3.1.4.1 Production system

The production systems, in which livestock is kept, may have a major impact on the risk for *T. gondii* infection (Table 6). However, the association of seropositivity in livestock within a particular production system provides no clear picture on the routes by which the animals became infected. Production systems are often related to specific conditions under which the animals are reared, fed or provided with water. These conditions may influence the likelihood of a contamination of feed, water or farmland with oocysts of *T. gondii* and the possibility of contact with the matrices mentioned above or with other infected intermediate hosts, e.g. rodents. In an intensive production system for example, the level of confinement is very high, at least for the respective livestock species, and thus, exposure of the animals to infective stages of the parasite is presumably lower as compared to extensive or other production system, where the animals have access to outdoor facilities. Intensive farming usually requires storing supplements. If these materials are stored open or in places where they may attract rodents or cats, additional routes of transmission may become relevant. Contaminated supplements may represent one explanation why intensive farming was not associated with a protective statistical effect in some studies (Table 6).

**Table 6:** Production system as a putative risk factor for *T. gondii* seropositivity in livestock.

Factor	Species	Statistically significant	Not statistically significant
Intensive	Pigs	9 (p), 20 (p), 28, 37 (p), 51 (p), 75 (p), 82 (p)	94
	Sheep	15 (p), 35 (p), 61, 69 (p), 81, 88 (p), 90 (p)	18, 57
	Goats	35 (p), 61, 68 (p), 81	–
Semi-intensive	Sheep	2, 35 (p), 68, 69, 81	36
	Goats	30, 35, 81	–
Extensive/animal friendly/organic/transhumance	Pigs	22, 51, 105	94
	Cattle	59, 78	21, 97
	Sheep	35, 58, 68, 88	14, 56
	Goats	35 (p), 68, 88	–
Backyard	Pigs	67	–
	Goats	47	–
	Chicken	4, 89, 91	–

(p) = protective factor; coding of references: 2 = Alvarado-Esquivel et al. (2013a), 4 = Alvarado-Esquivel et al. (2012a), 9 = Assadi-Rad et al. (1995), 14 = Condoleo et al. (2016), 15 = Cosendey-KezenLeite et al. (2014), 18 = de Barros Correia et al. (2015), 20 = de Sousa et al. (2014), 21 = de Souza et al. (2016), 22 = Deksne and Kirjusina (2013), 28 = Esteves et al. (2014), 30 = Garcia et al. (2012), 35 = Gazzonis et al. (2015), 36 = Gebremedhin et al. (2013), 37 = Gebreyes et al. (2008), 47 = Iovu et al. (2012), 51 = Kijlstra et al. (2004), 56 = Liu et al. (2015), 57 = Lopes et al., 2013, Lopes et al., 2013, 58 = Lopes et al. (2010), 59 = Magalhaes et al. (2016), 61 = Mainar et al. (1996), 67 = Pastiu et al. (2013), 68 = Rego et al. (2016), 69 = Rizzo et al. (2017), 75 = Schulzig and Fehlhaber (2005), 78 = Sun et al. (2015), 81 = Tzanidakis et al. (2012), 82 = van der Giessen et al. (2007), 86 = Villari et al. (2009), 88 = Xu et al. (2015), 89 = Xu et al. (2012), 90 = Zhang et al. (2016), 91 = Zhu et al. (2008), 94 = Samico-Fernandes et al. (2017), 97 = Fajardo et al. (2013), 105 = Wallander et al. (2016).

##### 3.1.4.2 Specific farming conditions

It was observed in several studies that differences in farming conditions, which are often livestock species-specific, were statistically significantly associated with differences in the risk of infection with *T. gondii* for various livestock species (Table 7). However, many of these associations are difficult to explain. Like production systems, livestock species-specific farming conditions may influence the probability of contamination of feed, water or farmland with *T. gondii* oocysts. This holds also true for other potential sources of exposure to *T. gondii*, e.g. the presence of infected intermediate hosts such as rodents. Specific conditions related to more extensive farming may also represent risk factors for seropositivity in livestock (Table 7).

**Table 7:** Livestock species-specific parameters as putative risk factors for *T. gondii* seropositivity.

Species	Factor	Statistically significant	Not statistically significant
Pigs	Complete production cycle performed on farm (farrow-to-finish)	17 (p), 34, 55 (p), 86 (p)	–
	Only part of production cycle performed on farm	17, 53, 55	–
	Origin of replacement sows, Source of pigs (own farm versus outside)	–	17, 55
Cattle	Mixed farming	–	78
	Beef farm (relative to dairy and mixed)	48 (p)	32, 78
	Feeder/stocker/backgrounder (versus feeder/stocker)	21	–
Sheep	Purpose subsistence (versus breeding/rebreeding/fattening)	69	–
	Purpose (meat, milk, mixed)	–	68
	Mixed (milk, meat)	14	–
Goats	Additional uses to dairy	26	–
	Purpose meat (versus genetic enhancement)	68	–
	Purpose (milk/meat/mix)	–	19, 68
Chicken	Breeders	89	–
	Layers	89	–
	Broilers	89 (p)	–
Equids	Racing	–	54
	Recreation	–	54
	Farming	54	–
	Use (breeding versus ceremonial, research or sports)^a^	101	–

(p) = protective factor; coding of references: 14 = Condoleo et al. (2016), 17 = Damriyasa et al. (2004), 19 = de Moura et al. (2016), 21 = de Souza et al. (2016), 26 = Djokic et al. (2014), 32 = Garcia-Bocanegra et al. (2013), 34 = Garcia-Bocanegra et al., 2010a, Garcia-Bocanegra et al., 2010b, 48 = Jokelainen et al. (2017), 53 = Klun et al. (2006), 54 = Kouam et al. (2010), 55 = Limon et al. (2017), 68 = Rego et al. (2016), 69 = Rizzo et al. (2017), 78 = Sun et al. (2015), 80 = Tao et al. (2011), 86 = Villari et al. (2009), 89 = Xu et al. (2012), 101 = Bartova et al. (2017).

a Statistics not conclusive.

##### 3.1.4.3 Herd and flock size

Herd or flock size is also related to the management and production system. Larger herds are more likely to be intensively managed. It can be assumed that farms with smaller herds often have a lower level of specialization and might be less confined. As farms applying animal welfare-oriented production techniques need more space to rear animals, this may restrict the size of the herd or flock. Again, herd or flock size may have an often un-explained link to conditions that can influence the probability of exposure of livestock to *T. gondii* due to contamination of feed, water or farmland with oocysts and contact to other infected intermediate hosts, e.g. rodents. There is a clear tendency showing that the smaller the herd or flock, the higher is the chance of seropositivity (Table 8).

**Table 8:** The effect of herd size on the seropositivity for *T. gondii* in livestock species.

Factor	Species	Statistically significant	Not statistically significant
Herd/flock size (numbers of animals as continuous variable)	Pigs	–	73
	Sheep	35, 46, 81	14, 32, 45, 56
	Goats	35 (p), 81 (p)	32, 56
	Cattle	–	78
	Equids	5	–
Small herd/flock size	Pigs	9, 25, 43, 55, 65, 86	
	Sheep	12, 36, 76, 88	95
	Goats	49, 88	95
	Cattle	38, 53, 95, 99 (p)	
	Chicken	93	
	Equids	104	102
Large flock size	Sheep	1, 18, 23	–
	Goats	1	
Higher number of sows	Pigs	25	–
Increased breeding density	Pigs	80	–
Dairy herds with a size ≥40–105 and >384 animals versus herds with a size <40 animals	Cattle	48	–
Mixed beef herd with a size of <50–200 animals versus herds with a size of >400 animals	Cattle	48	–
Flock size 150–300 versus >300 animals	Sheep	49 (p)	–
Low animal density in herd	Cattle	32	–

(p) = protective factor; coding of references: 1 = Abu-Dalbouh et al. (2012), 5 = Alvarado-Esquivel et al. (2012b), 9 = Assadi-Rad et al. (1995), 12 = Cenci-Goga et al. (2013), 14 = Condoleo et al. (2016), 18 = de Barros Correia et al. (2015), 23 = Deksne et al. (2017), 25 = Djokic et al. (2016), 32 = Garcia-Bocanegra et al. (2013), 35 = Gazzonis et al. (2015), 36 = Gebremedhin et al. (2013), 38 = Gilot-Fromont et al. (2009), 43 = Herrero et al. (2016), 45 = Holec-Gasior et al. (2015), 46 = Hutchinson et al. (2011), 48 = Jokelainen et al. (2017), 49 = Kantzoura et al. (2013), 53 = Klun et al. (2006), 55 = Limon et al. (2017), 56 = Liu et al. (2015), 65 = Ortega-Pacheco et al. (2013), 73 = Santoro et al. (2017), 76 = Sechi et al. (2013), 78 = Sun et al. (2015), 80 = Tao et al. (2011), 81 = Tzanidakis et al. (2012), 86 = Villari et al. (2009), 88 = Xu et al. (2015), 93 = Schares et al. (2017a), 95 = Tilahun et al. (2018), 99 = Schoonman et al. (2010), 102 = Machacova et al. (2014), 104 = Ribeiro et al. (2016).

##### 3.1.4.4 Presence of other animal species and contact to other herds

The presence of several animal species on a farm or other animals kept close to the livestock species may serve as an indicator of low farming intensity. Even contact to or mixing with other herds (of the same species, or from other farms) was established as a risk factor. As already discussed, low intensity was often related to a higher risk of *T. gondii* seropositivity (Table 6). The overall tendency that presence of or contact to other animal species may pose a risk for livestock to be *T. gondii*-seropositive (Table 9), could therefore be explained by the specific conditions in low-intensive farming. These conditions may be associated with an increased risk of exposure of livestock to *T. gondii* (e.g. through contamination of feed, water or farmland with oocysts and contact to rodents). On the other hand, the presence of other animal species could also have a direct effect on the risk of infection for livestock animals, as they may represent reservoirs for *T. gondii* and can thus be involved in the establishment of an on-farm multiplication of *T. gondii* (Table 9).

**Table 9:** Parameters related to the presence of other animals (livestock, non-livestock) close to livestock species as putative risk factors for seropositivity to *T. gondii*.

Species	Presence of other species, contact with other herds/flocks	Statistically significant	Not statistically significant
Pigs	Presence of dairy cattle	–	6, 25
	Presence of poultry	86	–
	Exposure to wild animals	–	25
	Other livestock	–	6, 25, 55
	Presence of dogs	34, 43, 86	6
Cattle	Presence of sheep/goats/pigs/poultry/canids; number of additional species on farm	97	78
Sheep	Contact with other flocks	50	14
	Grazing with other herds	49	18, 81
	Presence of different species	–	14
	Presence of animals (other than sheep) from other farms	35	14, 18
	Presence of wild animals	–	14
	Presence of cattle	22, 46	14
	Presence of goats	–	14, 35
	Presence of dogs	–	14, 68, 81
	Feeding of dogs with pet food (versus feeding food waste)	68	–
	Presence of wild dogs	–	68
	Presence of poultry	23 (p)	81
	Presence of pigs	–	14, 81
Goats	Common grazing with animals from other herds	49	81
	Presence of sheep	35	–
	Presence of dogs	–	81
	Number of dogs ≤2	68	–
	Feeding of the dogs with pet food/leftovers	–	68
	Dogs have access to water	68 (p)	–
	Dogs have access to facilities	–	68
	Presence of poultry	–	81
	Presence of pigs	–	81
	Presence of wild dogs	–	68
	Presence of other species	35, 96	–
	Solely goats on the farm	26	24
Chicken	Presence of sheep	71	–
	Birth of sheep on property	71	–
	Reproductive disorders in sheep	71	–
Equids	Presence of domestic ruminants	31	102

(p) = protective factor; coding of references: 6 = Alvarado-Esquivel et al. (2014), 14 = Condoleo et al. (2016), 18 = de Barros Correia et al. (2015), 23 = Deksne et al. (2017), 24 = Deng et al. (2016), 25 = Djokic et al. (2016), 26 = Djokic et al. (2014), 31 = Garcia-Bocanegra et al. (2012), 34 = Garcia-Bocanegra et al., 2010a, Garcia-Bocanegra et al., 2010b, 35 = Gazzonis et al. (2015), 43 = Herrero et al. (2016), 46 = Hutchinson et al. (2011), 49 = Kantzoura et al. (2013), 50 = Katzer et al. (2011), 55 = Limon et al. (2017), 68 = Rego et al. (2016), 71 = Sa et al. (2017), 78 = Sun et al. (2015), 81 = Tzanidakis et al. (2012), 86 = Villari et al. (2009), 96 = Bawm et al. (2016), 97 = Fajardo et al. (2013), 102 = Machacova et al. (2014).

##### 3.1.4.5 Biosecurity, farm buildings, staff hygiene, animal restocking

Biosecurity, the structural condition of farm buildings, staff hygiene and a restrictive policy of restocking seem to be associated with a lower risk of seropositivity in livestock (Table 10). In most cases, staff hygiene and biosecurity measures may have no direct effect on the exposure of animals to *T. gondii*, except for e.g. cleaning methods or the floor type (Table 10). However, the implementation of biosecurity and hygiene measures could represent an indirect indicator for the level of confinement under which livestock are reared. A restricted restocking policy by avoiding the purchase of animals from other farms may prevent the accidental incorporation of infected animals into the herd or flock.

**Table 10:** Parameters related to biosecurity, structural condition of farm buildings, staff hygiene and restricted animal restocking as putative risk factors for *T. gondii* seropositivity in livestock.

Species	Management, biosecurity and staff	Statistically significant	Not statistically significant
Pigs	Biosecurity	–	55
	Staff restriction	–	55
	Low level of staff hygiene	39	–
	Specialized boots, clothes	–	25, 43
	Proper maintenance of farm facilities	43 (p)	–
	Control of mosquitos and flies	34, 80	84
	Bird proof nets	34 (p)	–
	Removal of dead animals	84 (p)	–
	Floor type	–	17, 25, 84
	Farm holdings (one or more sites)	–	55
	Danish entry	25	–
Cattle	Presence of birds in stables	78	–
	Farm neighborhood (isolation)	38	–
	Work clothes available	–	78
	Slaughter on property	–	21
	Cattle introduced from other farms	78	–
	Dirty floor versus concrete floor	99	–
Sheep	State-owned farms (versus private)	53	–
	Commercial (versus family)	41	–
	Large size	70, 85	–
	Agriculture is main occupation	70	–
	Lambing in paddocks or parks	–	50
	Slatted floor	16, 68	–
	Presence of pen	–	41, 95
	Cement	68 (p)	–
	Dirt floor versus concrete floor	68	–
	Multiple boundaries	50	–
	Technified rearing	–	18
	Educational level of farmer	49	–
	Farm recently created	61	–
	Replacements during preceding year	61	18
	Use of exchanged or borrowed breeding males	16	–
	Leaving aborted fetuses on ground	1	81
	Predominantly external replacement	–	81
	Stocking rate (<1 versus ≥1)	41 (p)	–
	Use of quarantine	69 (p)	81
	Frequency of domestic slaughtering	–	14
	Availability of a special place for parturition	–	81
Goats	Pen flooring dirt (to suspended slat, mix, cement)	68	–
	Leaving aborted fetuses on ground	1	81
	Predominantly external replacement	–	81
	Purchase of spare breeding animals	–	35
	No replacements during preceding year	61	24
	Use of quarantine	–	81
	Availability of a special place for parturition	–	81
	Animals born on farm	26	–
	Educational level of farmer	49	–
	Farm recently created	61	–
Chicken	Intake of fetal adnexa, fluids and placentas	71	–
	Slaughter of animals on property	71	–
Equids	Animals of replenishments from other districts or states	29	–
	Use of studs from other stables	–	29
	Acquisition of female breeders in the last 5 years	–	29
	Introduced breeders in the last 5 years	–	29
	Treatment, cleaning and care area	104	–

(p) = protective factor; coding of references: 1 = Abu-Dalbouh et al. (2012), 14 = Condoleo et al. (2016), 16 = D'Alencar Mendonca et al. (2013), 17 = Damriyasa et al. (2004), 18 = de Barros Correia et al. (2015), 21 = de Souza et al. (2016); 24 = Deng et al. (2016), 25 = Djokic et al. (2016), 26 = Djokic et al. (2014), 29 = Fonseca de Araujo Valenca et al. (2015), 34 = Garcia-Bocanegra et al., 2010a, Garcia-Bocanegra et al., 2010b, 35 = Gazzonis et al. (2015), 38 = Gilot-Fromont et al. (2009); 39 = Goerlich (2011), 41 = Guimaraes et al. (2013), 43 = Herrero et al. (2016), 49 = Kantzoura et al. (2013), 50 = Katzer et al. (2011), 53 = Klun et al. (2006), 55 = Limon et al. (2017), 61 = Mainar et al. (1996), 68 = Rego et al. (2016), 69 = Rizzo et al. (2017), 70 = Romanelli et al. (2007), 71 = Sa et al. (2017), 78 = Sun et al. (2015); 80 = Tao et al. (2011), 81 = Tzanidakis et al. (2012), 84 = Veronesi et al. (2011), 85 = Vesco et al. (2007), 95 = Tilahun et al. (2018), 99 = Schoonman et al. (2010), 104 = Ribeiro et al. (2016).

##### 3.1.4.6 Hygiene, cleaning and disinfection measures

Measures of hygiene and regimes of cleaning and disinfection applied at the farms may play an important role in the infection of livestock with *T. gondii* because cleaning reduces the probability of contamination of the facilities with oocysts and may also reduce exposure to infected intermediate hosts, e.g. rodents. There is a clear tendency that a high hygienic status and the implementation of cleaning and disinfection measures has a protective effect (Table 11).

**Table 11:** Parameters related to hygiene, cleaning and disinfection measures as putative risk or protective factors for *T. gondii* seropositivity in livestock.

Species	Hygiene, disinfection and cleaning measures	Statistically significant	Not statistically significant
Pigs	Manual cleaning	86	–
	Frequency of disinfection	–	80
	Empty period length (short versus long)	39	25
	All-in/all-out	34 (p), 39 (p), 84 (p)	43, 55
	No cleaning and disinfection	34	–
	Cleaning method (only mechanical)	–	17
	Only removing manure	39	–
Cattle	Cleaning method	–	78
Sheep	Poor hygiene conditions	15, 56	–
	Hygiene level	–	81
	Disinfection of installations	69 (p)	–
	Use of dunghill	69 (p)	–
	Feces management	69 (p)	–
Goats	Feces management	–	68
	Poor hygiene level	56	81
Equids	No dunghill	29	–
Chicken	Service period prior to restocking	93	–

(p) = protective factor; coding of references: 15 = Cosendey-KezenLeite et al. (2014), 17 = Damriyasa et al. (2004), 25 = Djokic et al. (2016), 29 = Fonseca de Araujo Valenca et al. (2015), 34 = Garcia-Bocanegra et al., 2010a, Garcia-Bocanegra et al., 2010b, 39 = Goerlich (2011), 43 = Herrero et al. (2016), 55 = Limon et al. (2017), 56 = Liu et al. (2015), 68 = Rego et al. (2016), 69 = Rizzo et al. (2017), 78 = Sun et al. (2015), 80 = Tao et al. (2011), 81 = Tzanidakis et al. (2012), 84 = Veronesi et al. (2011), 86 = Villari et al. (2009), 93 = Schares et al. (2017a).

##### 3.1.4.7 Disease and treatment parameters and other factors characterizing herd health and veterinary care

Disease and treatment parameters and other factors characterizing herd health and veterinary care are often unrelated to *T. gondii* seropositivity in livestock (Table 12). Even when parameters characterizing reproductive problems in small ruminants were assessed, statistically significant associations were hardly observed. However, a few studies that had specifically looked at abortion in general, serial abortions or neonatal mortality, revealed associations to *T. gondii* seropositivity (Table 12).

**Table 12:** Disease and treatment parameters and other factors characterizing herd health and their association with *T. gondii* seropositivity in livestock.

Species	Factors	Statistically significant	Not statistically significant
Pigs	Deworming	–	6, 55
	Cannibalism	65	43
Cattle	Number of pregnancies (0 or 1 versus 3 or more pregnancies)	79	–
	Reproductive disorder	–	32, 21
	Brucellosis status	–	32
	Veterinary service	–	78
Sheep	No veterinary care	15	–
	Vaccination status (multivariable testing including age)	46	–
	Anthelmintic treatment	49 (p)	68
	Treatment with albendazoles (versus salicylanilides and imidazothiazoles)	49 (p)	–
	Previous history of serial abortions	61	–
	Unusual episodes of neonatal mortality	61	–
	Proportion of abortions (high versus low)	18	81
	Occurrence of stillbirths	–	18
	Occurrence of death at weaning	–	18
	Neurological problems observed	–	81
	Number of abortion waves per year	–	81
	Laboratory investigation of causes of abortion	–	81
	Reproductive disorder	15	32, 69, 81
	Brucellosis status	–	32
Goats	Proportion of abortions	–	81
	Neurological problems observed	–	81
	Number of abortion waves per year	–	81
	Laboratory investigation of causes of abortion	–	81
	Reproductive disorder	–	32, 81
	Brucellosis status	–	32
	Anthelmintic treatment	49	–
	Deworming	68 (p)	–
	Previous history of serial abortions	61	–
	Unusual episodes of neonatal mortality	61	–
Equids	Vaccination (tetanus, influenza)	104 (p)	–
	Use of embryo transfer	104 (p)	–

(p) = protective factor; coding of references: 6 = Alvarado-Esquivel et al. (2014), 15 = Cosendey-KezenLeite et al. (2014), 18 = de Barros Correia et al. (2015), 21 = de Souza et al. (2016); 32 = Garcia-Bocanegra et al. (2013), 43 = Herrero et al. (2016); 46 = Hutchinson et al. (2011), 49 = Kantzoura et al. (2013), 55 = Limon et al. (2017), 61 = Mainar et al. (1996), 65 = Ortega-Pacheco et al. (2013); 68 = Rego et al. (2016), 69 = Rizzo et al. (2017), 78 = Sun et al. (2015); 79 = Tan et al. (2015); 81 = Tzanidakis et al. (2012), 104 = Ribeiro et al. (2016).

### 3.2 Factors related to the *T. gondii* life cycle

#### 3.2.1 Definitive hosts (cats)

Cats as definitive hosts of *T. gondii* may shed oocysts with their feces. Excreted oocysts are environmentally resistant and become infective after a short period of sporulation. Thus, a large number of on-farm studies assessed cat-related factors in relation to seropositivity in livestock animals (Table 13). Surprisingly, about half of the studies which considered the presence of cats on the farms failed to find an association with seropositivity regarding *T. gondii*, especially in small ruminants, poultry and equids (Table 13). In addition, cat-related factors had a protective statistical effect in some investigations (Table 13). This shows that not just the presence of cats, but more specifically the realistic chance of cats to contaminate farmland, feed or water provided to livestock needs to be examined. A study in 12 pig farms in China indicated for example that the seroprevalence in pigs was higher in farms with a high cat density and with high soil contamination with *T. gondii* oocysts (as determined by PCR and loop-mediated isothermal amplification (LAMP)) than in those with low cat density (Du et al., 2012). If only a small number of pigs in the herd were infected, ingestion of tissue cysts, present in accidentally eaten intermediate hosts (e.g. birds or rodents), need to be taken into account as a potential source of infection. Although some research on serological methods to detect the source of infection (oocysts vs. cysts) in animals was reported (Hill et al., 2011; Munoz-Zanzi et al., 2012), such tools are hardly available or validated for epidemiological studies, unfortunately.

**Table 13:** Cat-related parameters as putative risk factors for *T. gondii* seropositivity in livestock.

Factor	Species	Statistically significant	Not statistically significant
Presence of cats on farm	Pigs	9, 20, 27, 33, 34, 39, 43, 65, 87	6, 17, 55, 84, 86, 94
	Cattle	38, 59, 78	21, 32, 38, 99
	Sheep	1, 13, 16, 56, 58, 61, 77, 85, 90	14, 15, 32, 35, 36, 41, 72, 81
	Goats	1, 61, 24, 56, 96	32, 35, 81
	Chicken	60, 93	71
	Equids	102	11, 31, 40, 29
Number of cats on farm (as continuous variable or >2–3 cats/>2/>3)	Pigs	27, 39, 63, 65	–
	Cattle	59, 97	–
	Sheep	41, 68	–
	Goats	24	68
	Chicken	60	–
Contact/exposure to cats or cat feces (plus frequency of exposure)	Pigs	63, 80	55
	Sheep	59	95
	Goats	19, 30, 68 (p)	24, 95
	Cattle	–	21, 95
Contact of cats with feed	Pigs	55	–
	Cattle	59	–
	Sheep	69, 70	68
	Goats	30	–
	Equids	102	29
Contact of cats with water	Pigs	–	55
	Sheep	12, 76, 68	41, 69
	Goats	68 (p)	–
	Equids	–	102
Vaccination of cats on farm	Pigs	62 (p)	–
Detection of oocysts in soil, cat feces, water	Pigs	27, 87	–
Cat-proof storage of feed supplements	Sheep	–	81
	Goats		81
Cats seen in hay	Sheep	–	81
	Goats	–	81
Cats are used for rodent control	Cattle	97	–
	Chicken	93	–
Population control of cats	Sheep	68 (p)	–
	Goats	–	68
Feeding of cats with pet food (versus food waste)	Sheep	68	–
	Goats	–	68
Cats feed on placenta remains	Sheep	–	68
	Goats	68	–
Presence of wild cats	Sheep	68 (p)	–
	Goats	68	–
Stray cats or wild felids on farm or farm land	Sheep	41	–
Occurrence of birth of cats on property	Chicken	–	71

(p) = protective factor; coding of references: 1 = Abu-Dalbouh et al. (2012), 6 = Alvarado-Esquivel et al. (2014), 9 = Assadi-Rad et al. (1995), 11 = Cazarotto et al. (2016), 12 = Cenci-Goga et al. (2013), 13 = Andrade et al. (2013), 14 = Condoleo et al. (2016), 15 = Cosendey-KezenLeite et al. (2014), 16 = D'Alencar Mendonca et al. (2013), 17 = Damriyasa et al. (2004), 19 = de Moura et al. (2016), 20 = de Sousa et al. (2014), 21 = de Souza et al. (2016), 24 = Deng et al. (2016), 27 = Du et al. (2012), 29 = Fonseca de Araujo Valenca et al. (2015), 30 = Garcia et al. (2012), 31 = Garcia-Bocanegra et al. (2012), 32 = Garcia-Bocanegra et al. (2013), 33 = Garcia-Bocanegra et al. (2010a), 34 = Garcia-Bocanegra et al. (2010b), 35 = Gazzonis et al. (2015), 36 = Gebremedhin et al. (2013), 38 = Gilot-Fromont et al. (2009), 39 = Goerlich (2011), 40 = Guerra et al. (2018), 41 = Guimaraes et al. (2013), 43 = Herrero et al. (2016), 55 = Limon et al. (2017), 56 = Liu et al. (2015), 58 = Lopes et al. (2010), 59 = Magalhaes et al. (2016), 60 = Magalhães et al. (2016), 61 = Mainar et al. (1996), 62 = Mateus-Pinilla et al. (1999), 63 = Meerburg et al. (2006), 65 = Ortega-Pacheco et al. (2013), 68 = Rego et al. (2016), 69 = Rizzo et al. (2017), 71 = Sa et al. (2017), 72 = Sakata et al. (2012), 76 = Sechi et al. (2013), 77 = Skjerve et al. (1998), 78 = Sun et al. (2015), 80 = Tao et al. (2011), 81 = Tzanidakis et al. (2012), 84 = Veronesi et al. (2011), 85 = Vesco et al. (2007), 86 = Villari et al. (2009), 87 = Weigel et al. (1995), 90 = Zhang et al. (2016), 93 = Schares et al. (2017a), Samico-Fernandes et al. (2017), 95 = Tilahun et al. (2018), 96 = Bawm et al. (2016), 97 = Fajardo et al. (2013), 99 = Schoonman et al. (2010), 102 = Machacova et al. (2014).

Studies that looked in more detail at the routes, by which cats could expose livestock to *T. gondii*, detected more frequently statistically significant associations. In addition, the chance to find a significant association was increased, when the number of cats present on a farm was taken into account (Table 13).

#### 3.2.2 Other on-farm intermediate hosts, such as rodents and their control

Rodents like mice and rats are intermediate hosts of *T. gondii* and may serve as a reservoir for the parasite on-farms. Cats, if allowed to ingest rodents that carry tissue cysts of *T. gondii*, may become infected and eventually shed oocysts. Omnivorous animals like pigs are at risk of getting infected through rodents. Overall, the recorded studies mainly showed that the presence of rodents and the absence of rodent control pose a risk for livestock to be *T. gondii*-seropositive (Table 14).

**Table 14:** Parameters related to intermediate hosts (rodents) and their control on farms as putative risk factors for *T. gondii* seropositivity in livestock.

Factor	Species	Statistically significant	Not statistically significant
Presence of rodents (mice, rats)	Pigs	–	87
	Cattle	78	–
	Sheep	15	–
Access of rodents to feed	Cattle	59	–
	Sheep	70	–
No rodent control measures	Pigs	9, 34, 43, 52, 86	51
	Sheep	77	–
Cats and dogs as a measure of rodent control/cats used for rodent control	Pigs	39, 44	–
	Chickens	93	–
Problems with mice and rats	Goats	–	24

Coding of references: 9 = Assadi-Rad et al. (1995), 15 = Cosendey-KezenLeite et al. (2014), 24 = Deng et al. (2016), 34 = Garcia-Bocanegra et al., 2010a, Garcia-Bocanegra et al., 2010b, 39 = Goerlich (2011), 43 = Herrero et al. (2016), 44 = Hill et al. (2010), 51 = Kijlstra et al. (2004), 52 = Kijlstra et al. (2008), 59 = Magalhaes et al. (2016), 70 = Romanelli et al. (2007), 75 = Schulzig and Fehlhaber (2005), 77 = Skjerve et al. (1998), 78 = Sun et al. (2015), 86 = Villari et al. (2009), 87 = Weigel et al. (1995), 93 = Schares et al. (2017a).

#### 3.2.3 Feed-related parameters

The uptake of infective stages of *T. gondii* with animal feed represents an important route, by which animals can get infected. Feed-related parameters are also influenced by the production system, which is in place on the farm. The evaluated studies suggest that open or less confined feed storage or feeding area represent an increased risk for exposure of livestock to the parasite (Table 15). In addition, feeding particular materials like goat whey may pose an infection risk as shown for pigs (Table 15). This suggests that goats excrete viable *T. gondii* stages in their milk, that may remain infective even after the whey has been produced (Table 15).

**Table 15:** Feeding-related parameters as putative risk factors for *T. gondii* seropositivity in livestock.

Species	Feeding characteristics	Statistically significant	Not statistically significant
Pigs	Food storage open	39	55, 65
	Food storage in owner's home	6	–
	Roughage not covered	63	–
	Manual feeder type (versus automatic feeder)	39	25, 55, 65
	Fluid feed (versus dry feed)	39 (p)	–
	Trough	39	–
	Feeding human food	6, 20	–
	Ration, mixed versus human food waste	–	94
	Feeding goat whey	63	–
Cattle	Use of silage	38	53
	Raw milk consumption	–	21
Sheep	Food storage uncovered	69	–
	Food through covered trough	69 (p)	–
	Feeding concentrate	81	–
	No mineral supplementation	58, 70	18
	Food source common	–	41
	Atypical grazing	77	–
Goats	Feeding concentrate	35, 81	19
	Use of mixer feeder	–	24
	Use of silo	–	24
Chicken	Feeding from ground	4	–
	Poultry feed indoors	4 (p)	–
Equids	Feed (with or without supplements)	–	40
	Mix of collective and individual troughs	29 (p)	–
	Ration consumption	–	29
	Ration storage location open	29	–
	Hay consumption	29	–
	Hay storage location open	29	–

(p) = protective factor; coding of references: 4 = Alvarado-Esquivel et al. (2012a), 6 = Alvarado-Esquivel et al. (2014), 18 = de Barros Correia et al. (2015), 19 = de Moura et al. (2016), 20 = de Sousa et al. (2014), 21 = de Souza et al. (2016), 24 = Deng et al. (2016), 25 = Djokic et al. (2016), 29 = Fonseca de Araujo Valenca et al. (2015), 35 = Gazzonis et al. (2015), 38 = Gilot-Fromont et al. (2009), 39 = Goerlich (2011), 40 = Guerra et al. (2018), 41 = Guimaraes et al. (2013), 53 = Klun et al. (2006), 55 = Limon et al. (2017), 58 = Lopes et al. (2010), 63 = Meerburg et al. (2006), 65 = Ortega-Pacheco et al. (2013), 69 = Rizzo et al. (2017), 70 = Romanelli et al. (2007), 72 = Sakata et al. (2012), 77 = Skjerve et al. (1998), 81 = Tzanidakis et al. (2012), 94 = Samico-Fernandes et al. (2017).

#### 3.2.4 Water-related parameters

Since oocysts of *T. gondii* can remain infective in water for a long time (i.e., under optimal conditions several months or even years (Dubey, 2010b)), it is hypothesized that the water supply for livestock may represent a risk factor for infection. Water can be supplied to the animals from a variety of sources such as tap water, wells or surface water and on different ways, which may depend on various factors like the production system or specific regional parameters. It is important to establish whether cats have access to the water at any stage before it reaches the livestock animals. Overall, from the recorded studies it is hard to quantify the risk for infection of the animals through contaminated water (Table 16). Often, the outcomes of the studies are contradicting. For example, well water was associated with an increased risk in some studies, while it seemed to have a protective statistical effect in others (Table 16).

**Table 16:** Water-related parameters as putative risk factors for *T. gondii* seropositivity in livestock.

Species	Water supply characteristics	Statistically significant	Not statistically significant
Pigs	Water supply (various sources assessed)	–	6, 55, 80, 84, 87
	Water from wells (versus municipal water)	86	–
Cattle	Water supply from pond or well	78	–
	Water point on pasture	38	–
	Water from reservoir	59	–
	Access to surface water	99	–
	Stagnant/pond water versus mixed water sources (river, stream, well)	95	–
	Tap water versus mixed water sources (river, stream, well)	95	–
Sheep	Use of surface water	85	
	Water from the public supply	81	41
	River water	36	41
	Tap water	36	–
	Water directly from the source (well)	15 (p), 16 (p)	69
	Still water (versus running water)	13 (p), 76	41
	Water from deep well	15, 16 (p)	–
	Water from sluice	15	–
	Stagnant/pond water versus mixed water sources (river, stream, well)	95	–
	Tap water versus mixed water sources (river, stream, well)	95	–
	Location of drinking trough	68	–
	Dogs and wild dogs have access to water	68	–
	Water sources in main grazing area	–	14
Goats	Location of drinking trough	68	
	Water source (various sources assessed)		19, 24
	Water from river	35	–
	Water from the public supply	81	–
	Stagnant/pond water versus mixed water sources (river, stream, well)	95	–
	Tap water versus mixed water sources (river, stream, well)	95	–
Chicken	Water source (dam)	60	–
Equids	Drinking from a mix of individual and collective troughs	29 (p)	–
	Water well versus public system	–	100
	Tank or river/stream versus public system	100	–

(p) = protective factor; coding of references: 6 = Alvarado-Esquivel et al. (2014), 13 = Andrade et al. (2013), 14 = Condoleo et al. (2016), 15 = Cosendey-KezenLeite et al. (2014), 16 = D'Alencar Mendonca et al. (2013), 19 = de Moura et al. (2016), 24 = Deng et al. (2016), 29 = Fonseca de Araujo Valenca et al. (2015), 35 = Gazzonis et al. (2015), 36 = Gebremedhin et al. (2013), 38 = Gilot-Fromont et al. (2009), 41 = Guimaraes et al. (2013), 55 = Limon et al. (2017), 59 = Magalhaes et al. (2016), 60 = Magalhães et al. (2016), 68 = Rego et al. (2016), 69 = Rizzo et al. (2017), 76 = Sechi et al. (2013), 78 = Sun et al. (2015), 80 = Tao et al. (2011), 81 = Tzanidakis et al. (2012), 84 = Veronesi et al. (2011), 85 = Vesco et al. (2007), 86 = Villari et al. (2009), 87 = Weigel et al. (1995), 95 = Tilahun et al. (2018), 99 = Schoonman et al. (2010), 100 = Almeida et al. (2017).

#### 3.2.5 Soil contact, outside access and pasturing

Exposure to contaminated soil or pastures seems to be another important factor (Table 17). Especially in pigs, extensive management or outdoor access seems to increase the chance for animals to get in contact with infective *T. gondii* stages (Table 17). Both, oocyst contaminations on farmland and, in the case of omnivorous livestock species, tissues of infected intermediate hosts represent likely sources of infection for livestock.

**Table 17:** Soil-contact, outside access, pasturing and related parameters as putative risk factors for *T. gondii* seropositivity in livestock.

Species	Soil-contact, outside access, pasturing	Statistically significant	Not statistically significant
Pigs	Outdoor facilities	6, 9, 22, 33, 53, 55	43
	Detection of oocysts in soil, cat feces, water	27, 87	–
	Scavenging	80	–
	Pasture length month	105	–
Sheep	Size of the grazing area	–	14
	Frequency of grazing	–	14
	Pasture	58	18, 41
Goats	Outdoor access	–	24
	Grazing	35	–
Cattle	Access to pasture (relative to stable only), grazing	53, 99	–
Equids	Shelter (in- or outdoor)	–	31, 40
	Pasture versus stable	101	–
Chicken	Size of chicken run per animal (≥10 sqm versus <10 sqm), multivariable analysis including age	93	–

Coding of references: 6 = Alvarado-Esquivel et al. (2014), 9 = Assadi-Rad et al. (1995), 14 = Condoleo et al. (2016), 18 = de Barros Correia et al. (2015), 22 = Deksne and Kirjusina (2013), 24 = Deng et al. (2016), 27 = Du et al. (2012), 31 = Garcia-Bocanegra et al. (2012), 33 = Garcia-Bocanegra et al., 2010a, Garcia-Bocanegra et al., 2010b, 35 = Gazzonis et al. (2015), 40 = Guerra et al. (2018), 41 = Guimaraes et al. (2013), 43 = Herrero et al. (2016), 53 = Klun et al. (2006), 55 = Limon et al. (2017), 58 = Lopes et al. (2010), 80 = Tao et al. (2011); 87 = Weigel et al. (1995), 93 = Schares et al. (2017a), 99 = Schoonman et al. (2010), 101 = Bartova et al. (2017), 105 = Wallander et al. (2016).

## 4 Economic impact of toxoplasmosis in livestock

In this review, we focused on articles that assessed the costs of *T. gondii* in livestock animals. It is important to stress, however, that *T. gondii* infections in animals used for food production may also affect human health and cause costs in this respect. These aspects are very difficult to assess and beyond the scope of the article, but have been addressed by others (Buzby and Roberts, 1997; Todd, 1989).

As summarized in Section 2.2, *T. gondii* is considered a major cause of reproductive losses in the small ruminant industry worldwide and infections in small ruminants may play a major role in the transmission of the parasite to humans (Belluco et al., 2016; Opsteegh et al., 2016). Especially in China, abortions caused by *T. gondii* in sows seem to be common and may lead to huge losses (Pan et al., 2017). There is one report on severe clinical signs in a fattening pig farm in China (Li et al., 2010) indicating that toxoplasmosis is of economic importance on these farms.

The number of studies assessing the economic impact of *T. gondii* infection in livestock is scarce. To estimate the economic consequences of an infection exclusively for the affected livestock species, i.e. leaving out any potential effect on human health, the clinical consequences, their severity and impact on the performance of the animals have to be analyzed. Small ruminants may suffer from a *T. gondii* infection and the infection can therefore cause economic losses to farmers. Experimentally infected sheep showed a number of clinical signs, which included fever, diarrhea, abortion, stillbirth, and fetal mummification (Dubey, 2009b). There is also one report indicating that *T. gondii* infected rams may become infertile (Savvulidi et al., 2018). It has also been suggested that *T. gondii* could be transmitted via semen (de Moraes et al., 2010; W.D. Lopes et al., 2013).

In general, the economic impact of diseases has different aspects that need to be taken into account. Direct costs of a disease (C) include not only losses (L), but also costs for the treatment of animals (T) and costs for disease prevention (P). The first aspect is ‘the value of the loss in expected output and/or of resource wastage due to the disease (L)’ (Bennett et al., 1999). In sheep, for example, lambs, wool, milk and meat represent the main output of a flock. As an example, in the case of a primary infection of sheep with *T. gondii*, there is a high probability of abortion (Dubey, 2009b) and the loss is therefore at least the value of the newborn lamb. Moreover, in dairy flocks, fever after acute infection, but mainly the occurrence of abortion, is related to the complete or partial loss of milk production for that lactation, i.e. the main source of income for these farms.

‘The costs for treatment of affected animals (T)’ represent the second aspect in an economic analysis (Bennett, 2003; Dijkhuizen and Morris, 1997). In the case of toxoplasmosis, the treatment costs include costs for anti-inflammatory substances to reduce fever or other veterinary services (e.g. removing mummified lambs, treatment of fertility problems after abortion, costs of laboratory diagnosis etc.).

‘The costs associated with specific disease prevention (P)’ form the third aspect in the economic analysis (Bennett et al., 1999). In the case of toxoplasmosis, vaccination of sheep on a regular base may represent such a preventive measure to reduce *T. gondii*-associated abortion.

There are only two formally published studies on the economic impact of toxoplasmosis in sheep and they refer only to two countries, Great Britain and Uruguay. Both studies focused on the losses that were due to abortion. Freyre et al. (1997), who analyzed the situation in Uruguay, estimated that about 10 million lambs were born in 1993 and that 14,000–400,000 sheep fetuses were aborted due to toxoplasmosis. They assumed a loss of 10 to 12 US $ per fetus, resulting in a loss of 1.4–4.68 million US $. They took neither the retardation of the genetic progress, nor the costs for replacement animals nor for husbandry into consideration.

Bennett and colleagues published several studies on the direct costs associated with endemic diseases of livestock in Great Britain, including toxoplasmosis in sheep (Bennett, 2003; Bennett et al., 1997, Bennett et al., 1999; Bennett and Ijpelaar, 2005). In the first study, referring to the year 1996, annual costs of 12–18 million £ for output losses (L) and <1 million £ for treatment (T), but no costs for prevention (P) were estimated. An incidence of ovine toxoplasmosis in ewes of 1.2 and 2.2% was assumed. In an update of this study, the authors estimated that about 334,000 (range: 136,000–526,000) sheep were affected per year, with disease effects of 9.1 (range: 3.7–14.1) million £ due to abortion or stillbirth and 3.2 (range: 2.7–5.6) million £ for disease control. It was assumed that toxoplasmosis caused 50,000 (range 8000–116,000) severely affected sheep in Great Britain per year. According to a recent ABC News report (https://www.abc.net.au/news/rural/2017-02-07/toxoplasmosis-costs-south-australian-sheep-producers/8245676, assessed on 11/12/2018, 12:32), a study carried out by Ryan O'Handley in South Australia in 2017 estimated toxoplasmosis costs in sheep at 70 million Australian $.

In summary, there are only a few formally published studies that assessed the costs of toxoplasmosis in livestock. They focused on sheep and peer-reviewed reports on this topic are >20 years old.

To improve the assessment of the economic impact of *T. gondii* on animals, it is necessary to carry out further studies including all aspects of the infection (Perry and Randolph, 1999) and all cost categories that can lead to losses. Information at the country level is scarce. Estimates are often based on studies conducted on (heavily) affected farms. This may lead to an overestimation of the total costs. It also remains unclear, if all recorded abortions have been caused by infection with *T. gondii*, even if part of the aborted fetuses has tested positive for toxoplasmosis. The lack of information on the effect of potential co-infections, e.g. border disease or bacterial infections that may also cause abortion, represents another factor of uncertainty.

In many publications with statements on costs the parameters included in the cost calculations remain unclear. It is therefore difficult to compare these analyses. Furthermore, they may include only costs directly related to the animals, but often ignore potential consequences on trade, a possible decrease of the value of infected flocks or herds and any potential impact on human health, if products contaminated with infectious stages of *T. gondii* are placed on the market. Consequently, we suggest that such studies list all parameters in a given livestock production system that might be affected by infection with *T. gondii* and indicate how they were taken into account in the cost calculations. As example, we provide such information categorized as direct and indirect costs in Table 18. It has to be noted, however, that some factors (e.g. herd management, hygiene) may have a general influence on the herd health status. This will make it hard to allocate any potential costs related to these factors to specific infections, for example with *T. gondii*.

**Table 18:** Factors possibly causing costs in livestock due to infection with *T. gondii*.

Type of costs	Costs	Comments
Direct costs (production losses caused by disease)	Reduced milk yield	Only in dairy sheep and dairy goats
	Weight loss in infected animals	Caused by fever and inappetence
	Reduced fertility	Cause of an increased replacement rate and retardation of the breeding progress
	Abortion/stillbirth	Cause of an increased replacement rate, loss of sells (e.g. lamb sells) and in retardation of the breeding progress
	Increased mortality	Reduced profit due to loss of animals and increased replacement rate
	Prolonged fattening periods in infected animals	Additional costs for feeding and reduced profit
	Weak or malformed progeny	Loss of progeny that causes additional costs, e.g. if a caesarian section has to be carried out or animals need veterinary service
Indirect costs (reaction to disease)	Optimization of herd management	Improvements in biosecurity, hygiene and farm buildings
	Treatment of diseased animals	Cost for veterinarian, drugs, etc.
	Control measures	For example vaccination
	Monitoring and diagnosis	For example costs for sampling and laboratory testing (also to achieve a differential diagnosis); testing of animals before housing
	Slaughter of infected animals	For example in animals with reduced fertility, increase in replacement costs
	Impact on trade (both national or international)	For example if meat of infected animals would be excluded from slaughter for specific meat products, or if international trade becomes restricted to avoid introduction of virulent types of *T. gondii*

## 5 Conclusion and future prospects

Although many studies on *T. gondii* infections and toxoplasmosis in livestock are available in literature, there are still several important gaps in our current knowledge. The routes of infection seem to be clear in herbivores, as oocysts can be presumed to be the main source. Nevertheless, there is uncertainty regarding many aspects, e.g. about the role of contamination of water and pastures and concerning the intensity of farming. In pigs, it seems clear that outdoor access is an important risk factor for *T. gondii* infection. It is not clear, however, to which extent oocysts contamination or the presence or uptake of infected intermediate hosts such as rodents play a role. Overall, further epidemiological studies are necessary and the standards in these studies need to be raised to obtain a better knowledge and more confidence regarding the epidemiologically relevant routes of *T. gondii* infection in livestock. In addition, more prospective studies including the assessment of interventions are needed to confirm previous findings and to determine the feasibility and the efficiency of control measures. Furthermore, the economic impact of toxoplasmosis in livestock, e.g. in small ruminants, has never been assessed in most of the regions worldwide, although especially small ruminants are economically important species in many countries. There is a clear need for further assessments of economic consequences of *T. gondii* infections and toxoplasmosis in livestock.

## Conflict of interest statement

None of the authors of this paper has a financial or personal relationship with other people or organizations that could inappropriately influence or bias the content of the paper.
